# A depression–like phenotype is associated with discrete defects in the primary hippocampal circuit

**DOI:** 10.1038/s41398-026-04094-3

**Published:** 2026-05-16

**Authors:** Benjamin G. Gunn, Chenyi C. Yang, Julie C. Lauterborn, Benedict S. Pruess, Julian Quintanilla, Katelyn Ge, Christine M. Gall, Gary Lynch

**Affiliations:** 1 Department of Anatomy and Neurobiology, University of California, Irvine, CA 92697 USA; 2 Department of Neurobiology and Behavior, University of California, Irvine, CA 92697 USA; 3 Department of Psychiatry and Human Behavior, University of California, Irvine, CA 92697 USA

**Keywords:** Hippocampus, Molecular neuroscience

## Abstract

Major depressive disorder is known to disturb the hippocampus, but how this impacts signal processing performed by the structure remains poorly understood. Here, we report that single housing (7-10 days) promotes a depression-like phenotype in young adult mice that is associated with a robust, yet surprisingly discreet defect in information flow across the primary hippocampal circuit. In addition to sociability disturbances and despair-like behavior, single housing eliminated preference for novelty and impaired episodic memory encoding. Additionally, the lateral habenula, an epithalamic structure critically involved in depression, was hyperactive. Although the CA1 waveform and associated spike output elicited by single-pulse lateral perforant path (LPP) activation of hippocampus was largely unaffected by single housing, pronounced disturbances emerged when the circuit was activated with physiologically relevant frequencies and patterns. The characteristic ‘theta/gamma’ pattern was distorted such that a pronounced facilitation was present in the single-housed group, while the filtering of CA1 output to brief beta (25 Hz) and gamma (50 Hz) frequency LPP stimulation evident in group-housed slices was absent. Within field CA3, the recruitment of inhibitory interneurons suppresses spike output, and subsequent signal propagation to CA1, in response to beta frequency LPP inputs but not those arriving at gamma frequencies. This CA3 beta filter was significantly impaired following single housing. These results suggest that a depression phenotype is associated with a highly selective and partial loss of inhibition within the CA3 and CA1 links of the hippocampal circuit, providing new insights into the relationship between depression and hippocampal function.

## Introduction

Major Depressive Disorder (MDD) is a prevalent and debilitating mental health disorder, associated with an extremely high personal and socioeconomic burden [1, 2]. While early work on the underlying pathophysiology focused on brainstem monoamine systems [3, 4], more recent clinical and preclinical studies point to perturbations in fast excitatory (glutamatergic) and inhibitory (GABAergic) transmission within cortical and limbic structures [5–7]. In particular, brain imaging studies, supported by postmortem analyses, have identified the hippocampus as one of the more severely disturbed forebrain regions in patients suffering from depression [8–13]. Recent experiments using transcranial magnetic stimulation to perturb forebrain circuits provided additional support for the hypothesis that a frontal-hippocampal network is centrally involved in MDD [14–19]. The hippocampus plays a central role in the processing of episodic or “everyday” memory [20] that provides the building blocks for higher cognitive functions. Notably, emotional content appears to promote long-term storage of episodes [21] and the hippocampus is well positioned to connect such information with signals arriving from the environment. The structure has extensive two-way connections with lower brain regions associated with emotions and with neocortical areas that underlie complex representations [22–24]. In all, hippocampal abnormalities provide a plausible explanation for why individuals experiencing depression exhibit i) deficits in episodic learning, ii) a tendency to preferentially remember experiences with a negative valence (i.e., memory bias), and iii) overgeneralize episodes [25].

Despite the above, little is known about how the development and progression of depression affects circuit level operation(s) within hippocampus. The absence of such information makes it difficult to specify the functional impairments introduced by depression, and relatedly, to localize cellular targets for treatment strategies. Of interest, clinical studies [12] and animal experiments [6, 7, 26] suggest that stress-related changes in the balance between excitatory and inhibitory (E/I) transmission is central to the onset and progression of depression. Pre-clinical models of depression [6, 7], many of which use stressor exposure (e.g., social, physical) as the inducing manipulation [6, 7, 27], have revealed functional changes in aspects of E/I transmission within specific hippocampal subfields [28–33]. Single housing of rodents, a mild, passive form of stress, promotes an anxious and depression-like phenotype when administered for prolonged periods (i.e., >2 weeks) or at specific developmental ages [34–39] and these behavioral effects are accompanied by alterations in synaptic transmission [40, 41]. Furthermore, manipulation of the GABAergic system, either through global reduction in γ2-GABA_A_R subunit expression or by lowering hippocampal and cortical GABA levels, has been associated with a depressive phenotype [42, 43]. While reasonable to assume that such alterations to synaptic transmission will produce sizeable disturbances to the processing of signals across the multiple links and nodes of the hippocampus, there have been no direct tests of this possibility.

In the work reported here, we first used behavioral and neurochemical approaches to establish that brief (7-10 days) single housing of adult mice produces diverse signs of a depression-like phenotype. These include a severe impairment to episodic memory, a characteristic feature of depression that is likely central to the cognitive problems associated with the disorder [7, 44, 45], as well as increased activity in the habenula, an epithalamic region that inhibits mesencephalic dopaminergic neurons and thereby depresses the generation of reward signals [45–48]. Hyperactivity in this nucleus is widely believed to be a major contributor to MDD [49, 50]. Second, we addressed the critical question of how hippocampal circuit operations are influenced during depressive episodes. Using a recently developed brain slice platform [51] we tested how single housing influences the CA1 spike output elicited by cortical inputs to the initial stages of the circuit. These data identified deficits in frequency-dependent signal transformations that are indicative of impaired inhibitory mechanisms in specific circuit elements.

## Materials and methods

A detailed description of animals and methods are included within the Supplementary Material. Animal protocols were registered and approved by the Institutional Animal Care and Use Committee (IACUC) at the University of California, Irvine.

### Animals

Studies used young adult (2-3 mo old) C57BL/6 male mice. Mice were group-housed (GH) with littermates (3-5 per cage), or single-housed (SH) for 7-10 days in the vivarium at 68 °F and 55% humidity with a 12 hr on/12 hr off light cycle and lights on at 6:30AM; food and water were provided *ad libitum*. Experimental mice were not previously handled.

### Behavioral assays

All behavioral tasks were video recorded using an overhead or lateral positioned camera (Logitech C920x HD Pro Webcam, 1080p/30fps). Videos were scored by two experimenters blind to group and mean score was used.

### Immunofluorescence & microscopy

#### Fos immunostaining

A subset of mice (GH and SH) were assessed for neuronal activity in the habenula using immunofluorescence for the cell activity marker Fos [52], 90 min after the conclusion of the Three-Chamber Task (i.e., after the recognition phase).

#### GABA_A_R subunit levels at inhibitory synapses

Mice (GH and SH) were euthanized and perfused, and brains processed for dual immunofluorescence for the scaffold protein gephyrin with specific GABA_A_R subunits (α2 and γ2) as described [53]. Fluorescence deconvolution tomography (FDT) [53–55] was used to quantify densities of immunolabeled GABA_A_R subunits at gephyrin-positive synapses in the apical dendrites of CA3b.

### Electrophysiological recordings

Hippocampal brain slices (400 µM) were prepared and an analysis of hippocampal circuit function was assessed from slices as previously described [51]. The spike output in response to lateral perforant path (LPP) activation, using single pulses and brief repetitive stimulation across a range of frequencies (θ [5 Hz], β [25 Hz] and γ [50 Hz]) and patterns (θγ), was recorded from the cell body layer of CA1 and CA3 of each group.

### Statistical analysis

Results are presented as group means ± SEM. Statistical significance was determined using GraphPad Prism (v6.0). Details of statistical analyses are presented in Supplementary Table 1.

## Results

### Behavioral consequences of single housing

**Fig. 1:**
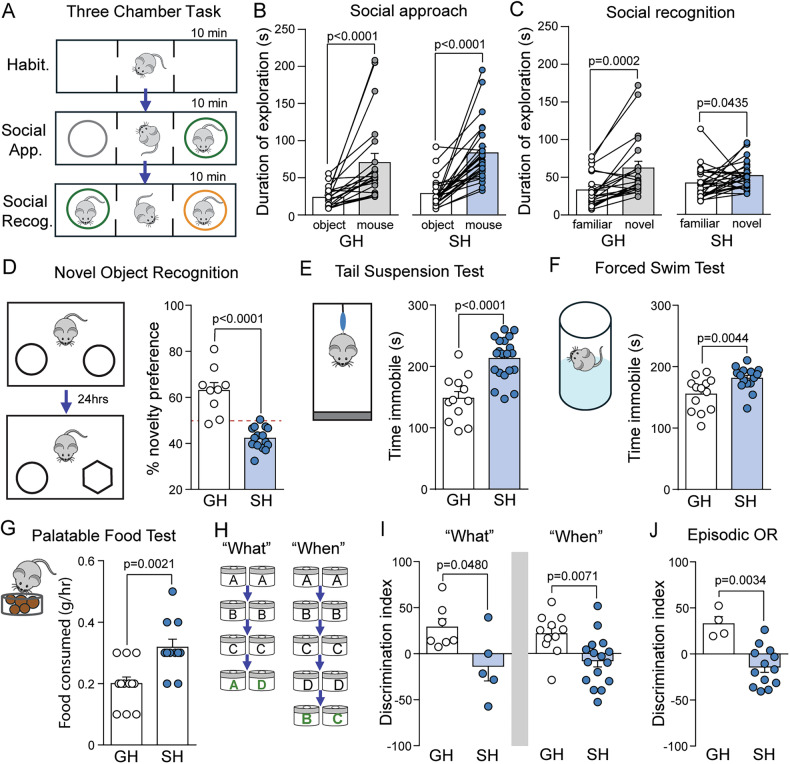
Behavioral consequences of single housing mice for 7-10 days (single-housed, SH) as compared to group-housed mice (GH) in the same vivarium colony. (**A**) Schematic depicting the Three-Chamber Task. The test animal is (i) habituated (Habit.) in an empty chamber for 10 min, (ii) exposed to an empty cup vs. mouse #1 (green circle) (social approach, App.), and (iii) exposed to novel mouse #2 (gold circle) vs. familiar mouse #1 (social recognition, Recog.). (**B,C**) Duration of time (seconds) exploring (**B**) the object or mouse in the social approach phase and (**C**) the familiar or novel mouse in the social recognition phase. (**D**) *Left*: Schematic of Novel Object Recognition paradigm. *Right*: Percent novelty preference for GH (avg = 62.99%) vs. SH (avg = 42.24%) mice with chance level illustrated (red line); the SH mice showed significantly less novelty preference. (**E,F**) Graphs show duration (seconds) of immobility of GH vs. SH mice in the (**E**) Tail Suspension Test and (**F**) Forced Swim Test; in each case the SH mice exhibited greater immobility. (**G**) Palatable Food Task. Amount of food (coco pebbles) consumed on the final (5th day) day is plotted for GH vs. SH. (**H**) Illustration of the serial cue, odor-based, episodic ‘What’ and ‘When’ memory tasks. (**I**) Discrimination indexes for GH vs SH mice in the odor-based ‘What’ (*left*) and ‘When’ (*right*) tasks show impaired acquisition by SH mice. (**J**) Discrimination indexes for episodic object recognition (OR) show impaired acquisition in SH vs GH mice. See Supplementary Table 1 for statistical details.

Consistent with earlier work [56–58], control, GH mice tested in the three-chamber social task (Fig. 1A) exhibited a significant preference for the mouse in the social approach phase (Fig. 1B), and spent considerably more time interacting with the novel (versus familiar) mouse in the recognition phase (Fig. 1C). This was not the case for SH mice. Despite showing a similar degree of preference for the mouse in the approach phase, SH mice did not differentiate between the familiar or novel mouse in the recognition phase (Fig. 1C). As anticipated, the time allocated to the mouse in the social approach phase did not differ between groups, whereas the difference in relative time allocated to the novel vs familiar mouse was significant (Supplementary Fig. 1A, B). Total exploration time during the social approach phase was modestly increased in the SH group, while there was no difference in the social recognition phase (Supplementary Fig. 1C, D).

The recognition phase of the protocol involves short-term memory (of the mouse encountered in the recent past), and it is possible that problems with encoding contribute to the absence of the normal preference in SH animals. Thus, we tested for memory deficits with the widely used novel object recognition test. While control GH mice displayed the anticipated strong preference for novel objects indicative of normal memory function, SH mice, somewhat surprisingly, expressed the opposite preference, spending more time with the familiar objects (Fig. 1D). The cue exploration times in the initial sampling period were comparable between groups, whereas clear differences were evident in the test phase (Supplementary Fig. 1E, F). We conclude that single housing leads to a reduced interest in novelty without generalized memory impairment.

The tail suspension and forced swim tests are commonly used to test for a depression-like syndrome in rodents. Both assays measure immobility time, with increased immobility times indicative of a depression-like phenotype. Immobility values for both tests were much higher in SH cases relative to GH, controls (Fig. 1E, F). Depression is often accompanied by increased or decreased consumption of palatable foods [59–61], with the cause generally assigned to abnormal perception of reward. Here, single housing produced a sizeable increase in consumption of sugary foods (Fig. 1G). In all, a relatively brief period of single housing caused multiple behavioral changes that align with clinical symptoms of depression.

MDD is associated with significant impairments to episodic memory, a type of encoding that is critically dependent upon hippocampus. We tested SH and GH mice for acquisition of two fundamental elements of an episode: the identities and temporal order for items in a collection of cues (respectively, ‘what’ and ‘when’; Fig. 1H). As with human episodic memory, the protocols involved first-time exposure to multiple cues without explicit rewards or repetition. To test for acquisition of ‘what’ information, mice were presented with a sequence of three odors (A:A > B:B > C:C) and then, after a brief delay, they were exposed to a cue that was previously sampled vs. one that was not (A:D; Fig. 1H***left***). GH mice showed a preference for the novel cue D, indicating that they remembered (recognized) cue A (Supplementary Fig. 2A). SH mice did not investigate cue D to a greater extent than cue A in the retention trial (Supplementary Fig. 2B). The discrimination scores for the two groups were markedly different (Fig. 1I***left***).

Time and sequencing of events are defining features of an episodic memory. We tested for this element by serially presenting the mice with four odors and then, during a retention trial, testing if they preferentially interact with cues according to position within the sequence (Fig. 1H***right***). GH mice spent considerably more time investigating an earlier cue (‘B’) than a later one (‘C’) when ‘B’ had preceded ‘C’ in a sequence (Supplementary Fig. 2B). This effect was not present in SH animals: there was no reliable difference in sampling time for B vs. C (Supplementary Fig. 2B). The discrimination index was substantially higher for the GH mice (21.3 ± 6.7%) than for the SH mice (-7.8 ± 6.8%, Fig. 1I***right***).

Finally, we conducted an episodic memory test that did not use odors as cues. Mice were given 5 min to explore a collection of four objects, and then after a 24-h delay, were again exposed to 4 objects substituting one initial object with a novel object. As expected from results for a similar task using odors [62], control mice spent considerably more time sampling the novel object than they did with the remaining three cues (Fig. 1J). This result indicates that the GH mice remembered the objects encountered during the initial exploration session. SH mice expressed no preference for the novel object, as expected if they had a impairment to episodic ‘what’ memory.

### Single housing activates the lateral habenula

**Fig. 2:**
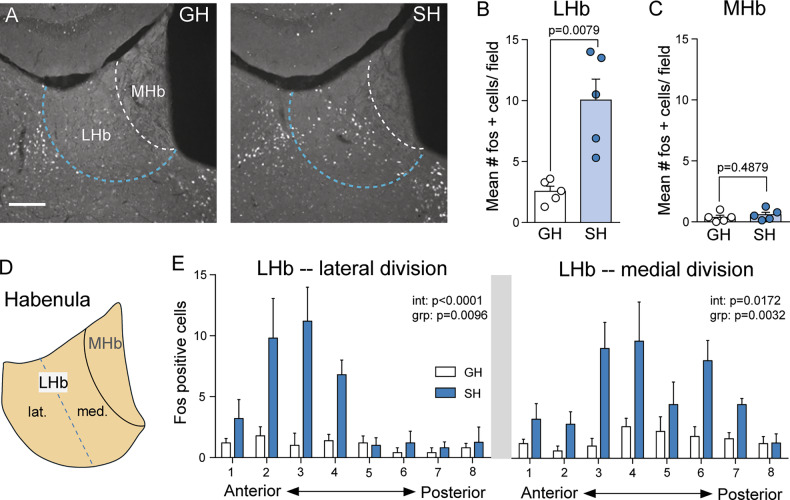
Single housing increases activity in lateral habenula. (**A**) Photomicrographs showing representative sections of the habenula with Fos immunopositive (+) neurons in grouped-housed (GH) and single-housed (SH) mice. The division between lateral (LHb) and medial (MHb) habenula is indicated by a white dotted line; the blue dotted line encompasses the LHb. Bar, 100 μm. (**B**) Numbers of Fos+ cells in LHb were greater in SH vs. GH groups, whereas (**C**) labeled cell counts were low in MHb of both SH and GH groups; mean ± SEM per sample field shown. (**D**) Schematic showing the lateral (lat.) and medial (med.) divisions of the LHb used for quantification in panel E. (**E**) Histograms show the anterior to posterior distribution of Fos+ cells (counts from individual tissue sections) in the lateral (*left*) and medial (*right*) LHb divisions for SH and GH mice (group mean ± SEMs): int, interaction effect; grp, group effect. See Supplementary Table 1 for statistical details.

The lateral habenula (LHb) inhibits dopaminergic neurons in the ventral tegmental area [63, 64]. The dopamine cell groups play a central role in mood control and, as might be expected from this, diverse lines of evidence indicate that elevated activity in the habenula leads to depression symptomatology [65–68]. Accordingly, we used Fos immunolabeling to test if the depression-like behavioral phenotype recorded in SH mice is accompanied by elevated numbers of active LHb neurons. Survey micrographs showed that labeling, 90 min after the conclusion of the three chamber task (i.e., after social recognition), was extremely sparse in LHb of GH mice but present at significant levels in SH mice (Fig. 2A); cell counts across all LHb fields were nearly four-fold greater in SH vs. GH mice (Fig. 2B; GH; 2.6 ± 0.4 cells/sampling zone, SH: 10.0 ± 1.7 cells /sampling zone). The pronounced effects of single housing in the LHb were not evident in the medial habenula, where labeled cells were rare in both groups (Fig. 2C).

Quantitative analyses of Fos immunolabeling were performed for 7-8 sections along the anterior-posterior dimension for both medial and lateral domains within the LHb (Fig. 2D) due to evidence that the two subfields have distinguishable input/output relationships [67, 69, 70]. Counts in both medial and lateral LHb subfields were much higher in SH than in GH mice. Labeling was not evenly distributed along the anterior-posterior axis (Fig. 2E) in the SH mice, with a strong tendency towards higher values in the anterior sections and in the lateral but not medial subfield. Collectively, these data suggest that single housing disrupts one or more inputs that regulate LHb activity and thereby increases descending inhibition of hindbrain systems that modulate mood and arousal. In any event, the results reinforce the conclusion that a week of single housing initiates a condition that resembles clinical depression.

### Effects of single housing on throughput in the primary hippocampal circuit

**Fig. 3:**
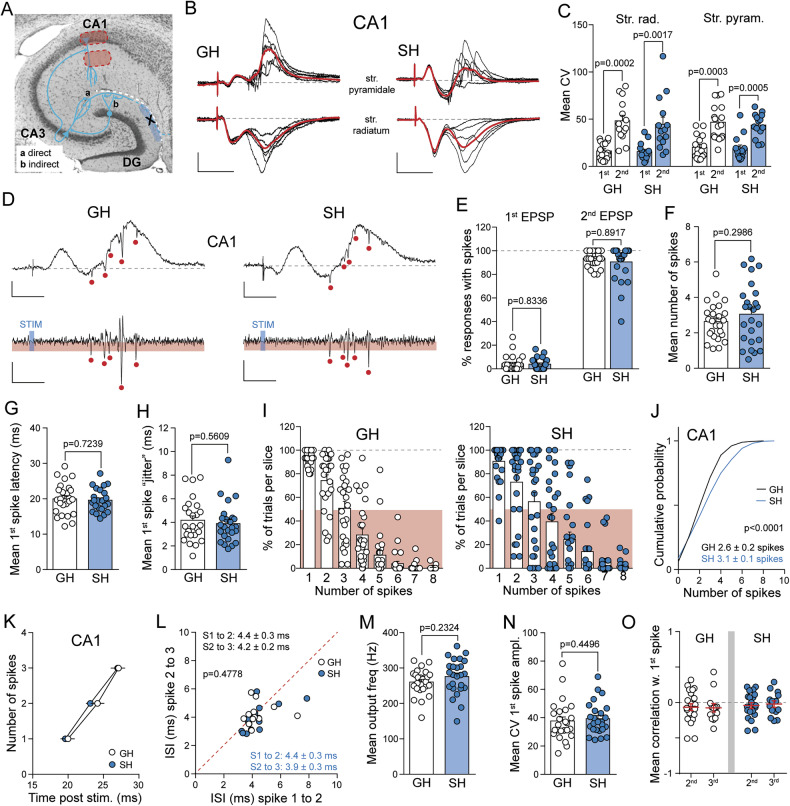
Single housing has no effect on CA1 responses to single pulse LPP activation. (**A**) Nissl stain of a hippocampal slice illustrating the location of the stimulating electrode (x, blue field) in the LPP field within the dentate gyrus outer molecular layer and two recording pipettes in CA1 (red fields). The composition of the hippocampal circuit is depicted with the direct (a) and indirect (b) sub-circuits shown. (**B**) Representative LPP-evoked two-part fEPSP recorded from CA1 str. pyramidale and str. radiatum of slices from GH (*left*) and SH (*right*) mice. The ensemble average fEPSP for each is shown in red (scale bars: y = 0.25 mV, x = 20 ms). (**C**) Graph shows the mean variance (CV) associated with the initial rising slope of the 1^st^ and 2^nd^ components of the response recorded from the CA1 dendritic field (Str. rad.; *left*) and pyramidal cell layer (Str. pyram.; *right*): No difference was observed between GH and SH slices. (**D**) Exemplar raw (*top*) and filtered (*bottom*) CA1 responses to single-pulse LPP stimulation recorded from GH (*left*) and SH (*right*) slices. Red dots identify single units in the raw and filtered trace for each (scale bars: y = 0.25 mV (top) and 0.1 mV (bottom), x = 10 ms for both). (**E**) Graph showing the percent (%) of CA1 responses exhibiting single units in the initial (1^st^ *EPSP*) and second (2^nd^ *EPSP*) component of the waveform did not differ between GH and SH slices. (**F**-**H**) Graphs illustrating the mean number of CA1 spikes (**F**), the latency to first spike (**G**) and the associated ‘jitter” (**H**) in response to single-pulse LPP stimulation in GH and SH slices. **(I)** Graphs show the percent (%) of LPP-evoked CA1 responses containing 1 to 8 spikes for GH (*left*) and SH (*right*) slices. Points show individual evoked response values; bars show mean ± SEMs. (**J**) Cumulative probability plot shows the difference in the distribution of spike numbers per response recorded from GH (738 trials) and SH (710 trials) slices. (**K**) Graph shows the mean number of spikes and their temporal distribution within the LPP-evoked CA1 response in GH and SH slices. (**L**) Scatter plot with unity line (red dashed) shows the relationship between the interspike interval (ISI) between the 1^st^ and 2^nd^ spikes and the 2^nd^ and 3^rd^ spikes in the LPP-evoked CA1 response in GH and SH slices. (**M,N**) Graphs show the mean output frequency of LPP-evoked CA1 spikes (**M**) and the mean variance (CV) of the 1^st^ spike amplitude (**N**) for each GH and SH slice. (**O**) Graph shows the mean correlation of the amplitude of the 1^st^ spike with the amplitudes of the 2^nd^ and 3^rd^ spikes in CA1 after single-pulse LPP stimulation in GH and SH slices. See Supplementary Table 1 for statistical details.

To test for SH-related disturbances to hippocampal operations, we used a recently described hippocampal slice preparation that samples throughput across the entirety of the primary circuit from cortical input to CA1 cell spiking (Fig. 3A; see [51]). Single-pulse stimulation of LPP projections from lateral entorhinal cortex (EC) produced a two-part field excitatory postsynaptic potential (fEPSP) in CA1 of slices from GH and SH mice (Fig. 3B). The initial and secondary components were negative in the proximal apical dendrites and positive in the cell body layer, indicating that the fEPSPs were generated by the dense projection from CA3 to CA1 str. radiatum (Fig. 3B). The second of the two waveforms displayed significantly greater variance (Fig. 3C) and was associated with the great majority of CA1 spiking elicited by LPP-activation: Both features were unaffected by single housing (Fig. 3C-E). Notably, the position of the stimulation electrode in the outer molecular layer of the dentate gyrus (DG; Fig. 3A) ensures that the direct temporoammonic projection (LPP-CA1) from layer III EC was not activated.

Single housing had subtle effects on CA1 spike output in response to single-pulse LPP activation. The mean number of evoked spikes, and the associated variability within and across slices, was virtually identical between GH and SH groups (Fig. 3F, Supplementary Table 2). The latency and “jitter” for the first spike were also not detectably different between groups, and the reliability and number of evoked spikes appeared unaffected by single housing (Fig. 3G-I, Supplementary Table 2). However, plotting the number of evoked spikes elicited by individual trials from all slices for each group as a cumulative probability revealed a rightward shift in the SH curve relative to the GH curve (Fig. 3J). Despite such variability in spike number, the inter-spike interval (ISI) for evoked responses was remarkably consistent across slices from each group (Fig. 3K). The intervals between the 1^st^ and 2^nd^ spikes and the 2^nd^ and 3^rd^ spikes were virtually identical for the two groups (Fig. 3L): a feature that resulted in a high CA1 output frequency (Fig. 3M). This high output frequency, when considered with the within-slice variability of the 1^st^ spike amplitude and lack of correlation of the 1^st^ spike amplitude with those of successive spikes (Fig. 3N, O), strongly suggests the LPP stimulation elicits near synchronous activation of a small number of CA1 pyramidal cells.

Collectively, these observations indicate that the depression-like phenotype induced by single housing had minimal effects on hippocampal circuit excitability in response to single-pulse LPP activation.

### Frequency-dependent circuit operations are impaired by single housing

**Fig. 4:**
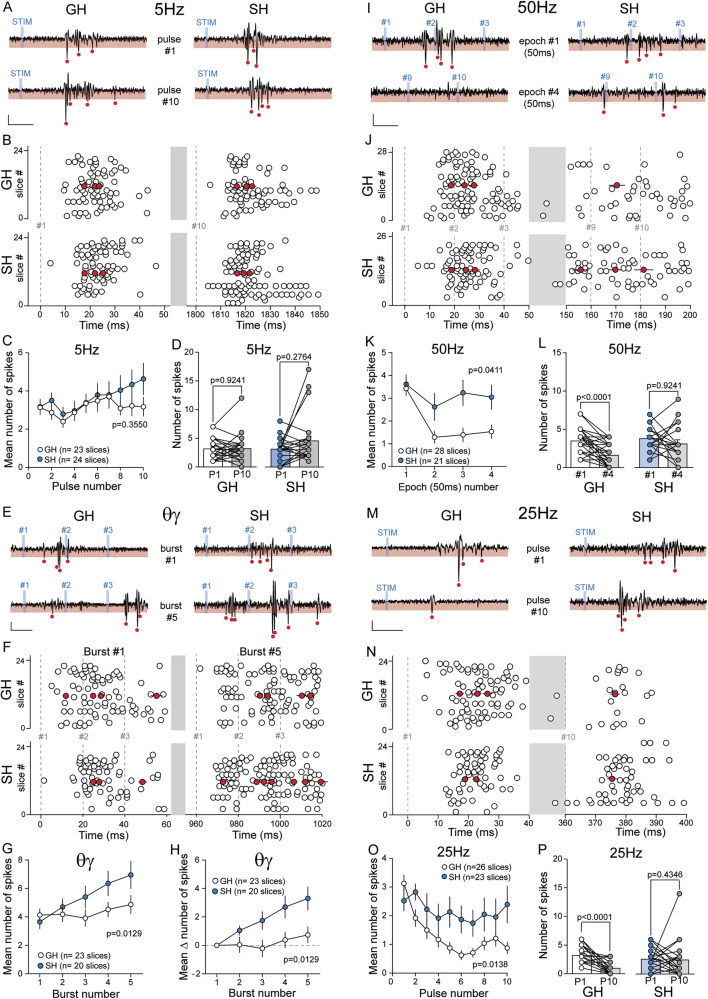
Single housing selectively impairs low pass filtering within the hippocampal circuit. **(A)** Representative filtered LPP-evoked CA1 responses show the spikes (red dots) elicited by the 1^st^ (*top*) and 10^th^ (*bottom*) pulse of a 5 Hz (10 pulse) stimulation train (STIM, stimulation) in slices from GH (*left*) and SH (*right*) mice (scale bars: y = 50 μV, x = 10 ms). (**B**) Raster plots show the distribution of individual CA1 spikes across time in response to the 1^st^ (*left*) and 10^th^ (*right*) stimulation pulse for each slice (open circles; y axis) from GH (*top*) and SH (*bottom*) mice; red circles indicate the mean time of CA1 spikes for all slices in each group. (**C**, **D**) Graphs summarizing the mean number of LPP-evoked CA1 spikes for each pulse of a 5 Hz stimulation train (**C**) and the number of spikes elicited by the 1^st^ and 10^th^ pulse (**D**) for each group. (**E**) Representative filtered LPP-evoked CA1 spike response to the 1^st^ (*top*) and 5^th^ (*bottom*) burst of a theta-gamma (5 bursts) stimulation train in GH (*left*) and SH (*right*) slices (scale bars: y = 50 μV, x = 10 ms). (**F**) The distribution of CA1 spiking across time in response to the 1^st^ and 5^th^ burst for each slice in both groups; red circles indicate the mean distribution of CA1 spikes for all slices in each group. (**G**, **H**) Graphs summarize the mean number of evoked spikes (**G)** and the mean change in the number of evoked spikes (**H**) across successive bursts during theta-gamma LPP stimulation in GH and SH slices. **(I**) Representative filtered LPP-evoked CA1 spike response during the 1^st^ (*top*) and 4^th^ (*bottom*) 50 ms epoch of a 50 Hz (10 pulse) stimulation train in GH (*left*) and SH (*right*) hippocampal slices (scale bars: y = 50 μV, x = 10 ms). (**J**) The distribution of individual CA1 spikes during the 1^st^ (*left*) and 4^th^ (*right*) 50 ms epoch illustrated for each group (GH: *top*; SH: *bottom*); red circles indicate the mean distribution of CA1 spikes for all slices in both groups. (**K**, **M**) Graphs summarize the mean number of LPP-evoked spikes for each of the 50 ms epochs of a 50 Hz stimulation train (**K**) and the number of spikes elicited during the 1^st^ and 4^th^ 50 ms epoch (**L**) for each group. **(M**) Representative filtered LPP-evoked CA1 spike responses during the 1^st^ (*top*) and 10^th^ (*bottom*) pulse of a 25 Hz stimulation train in slices from GH (*left*) and SH (*right*) mice (scale bars: y = 50 μV, x = 10 ms). (**N**) The distribution of CA1 spiking during the 1^st^ and 10^th^ stimulation pulse are illustrated for each group (GH: *top*; SH: *bottom*); red circles indicate the mean distribution of CA1 spikes for all slices in both groups. (**O**, **P**) Graphs summarize for each group the mean number of LPP-evoked spikes for each pulse of a 10- pulse, 25 Hz stimulation train (**O**) and the number of LPP-evoked spikes for each slice elicited by the 1^st^ and 10^th^ pulse of the train (**P**). Group results are mean ± SEM values; see Supplementary Table 1 for statistical details.

Communication in the cortical telencephalon depends on rhythmic activity, with different frequency ranges or patterns associated with particular behavioral activities. The hippocampal circuit operates as a low pass filter: LPP activation with brief theta (5 Hz; 10 pulses) or theta-gamma (5 bursts) stimulation trains drive signal throughput, whereas inputs delivered at gamma (50 Hz) frequencies are heavily filtered [51]. Thus, we first tested if single housing influenced signal throughput. LPP stimulation at 5 Hz elicited a CA1 spike output that was consistent across a 10-pulse train in slices from GH and SH mice (Fig. 4A, B). In both groups, LPP-evoked CA1 spiking was present on almost all pulses within the train (Fig. 4B, C) with minimal transformation of this output between the 1^st^ and last pulse (Fig. 4B-D). Theta-gamma stimulation, a pattern that obviates the low pass filter, produced a reliable CA1 spike output in the GH group that displayed minimal transformation(s) across successive bursts (Fig. 4E, F). Surprisingly, following single housing, theta-gamma activation of LPP inputs produced CA1 spiking that was amplified across successive bursts (Fig. 4E, F). The mean number of spikes and the change in spike numbers per burst increased significantly across the theta-gamma train in the SH group only (Fig. 4F-H). These observations indicate that single housing has no effect on signal throughput in response to 5 Hz stimulation, while the apparent amplification of CA1 spiking across successive theta-gamma bursts suggests potential defects in mechanism(s) required to curtail the activity elicited by gamma frequency bursts.

To investigate the possibility that single housing produces deficits in low pass filtering across the hippocampal circuit, we measured the CA1 spike response to brief 50 Hz LPP stimulation. Slices from GH mice exhibited a clear decrease in CA1 spiking across the 50 Hz train, an effect that was attenuated by single housing (Fig. 4I, J). Quantification of LPP-evoked CA1 spikes revealed a significant reduction between the first and fourth 50 ms epoch in the GH group that was almost completely absent in the SH group (Fig. 4K, L). A subsequent set of experiments investigated the nature of the LPP-evoked CA1 spike output in response to brief beta (25 Hz) frequency stimulation. The CA1 spiking elicited during the stimulation train was again heavily filtered in the GH group, and this effect was severely impaired in slices from SH mice (Fig. 4M-P).

Overall, these findings indicate that exposure to single housing produces perturbations within the hippocampal circuit that result in impaired filtering of signals arriving at beta frequencies and higher.

### Location of defective circuit elements in slices from SH mice

The LPP projections utilize direct (LPP-CA3-CA1) and indirect (LPP-DG-CA3-CA1) routes to drive throughput to CA1 [71–73]. The indirect path engages the CA3 recurrent collateral system, and this proves to be critical for activating robust CA1 spike output [51]. We tested if the defective filtering in SH mice relates to problems distributed across this complex system of connections or if instead it is due to discrete failures at specific links.

The LPP-DG synapse operates as a potent low pass filter in that responses to gamma frequency inputs are suppressed while those arriving at theta and beta are not [74]. Defects at this first stage could therefore account for the abnormal CA1 spike output elicited by gamma frequency activation of the LPP after single housing. However, LPP-DG responses to a gamma train were not different between GH and SH mice (Supplementary Fig. 3A-D). Synaptic and population spike responses evoked by beta and theta trains were also comparable between groups (Supplementary Fig. 3E-H).

Next, we tested for SH-related alterations in field CA3. Single-pulse LPP stimulation elicited responses in the CA3a cell body layer (str. pyramidale) comprised of a modest but temporally extended fEPSP (0.5-1 mV amplitude) that contained a large number of single units (4-10 spikes; Supplementary Fig. 4A-C). The mean number and frequency of LPP-evoked spikes, as well as the latency to first spike and associated “jitter” were not different between groups (Supplementary Fig. 4D-G & Supplementary Table 3). The amplitude of the 1^st^ spike was not correlated with those for any of the subsequent spikes, suggesting that the response did not involve single pyramidal cells (Supplementary Fig. 4H-J & Supplementary Table 3). The distribution of the number of spikes elicited by LPP stimulation was modestly shifted to the right (Supplementary Fig. 4K, L). As with the CA1 spike output, the duration and variability for the mean ISI were remarkably similar for the two groups (Supplementary Fig. 4M-P**)**, indicating that the self-organizing properties of the CA3 recurrent collateral system were largely unaltered by single housing.

**Fig. 5:**
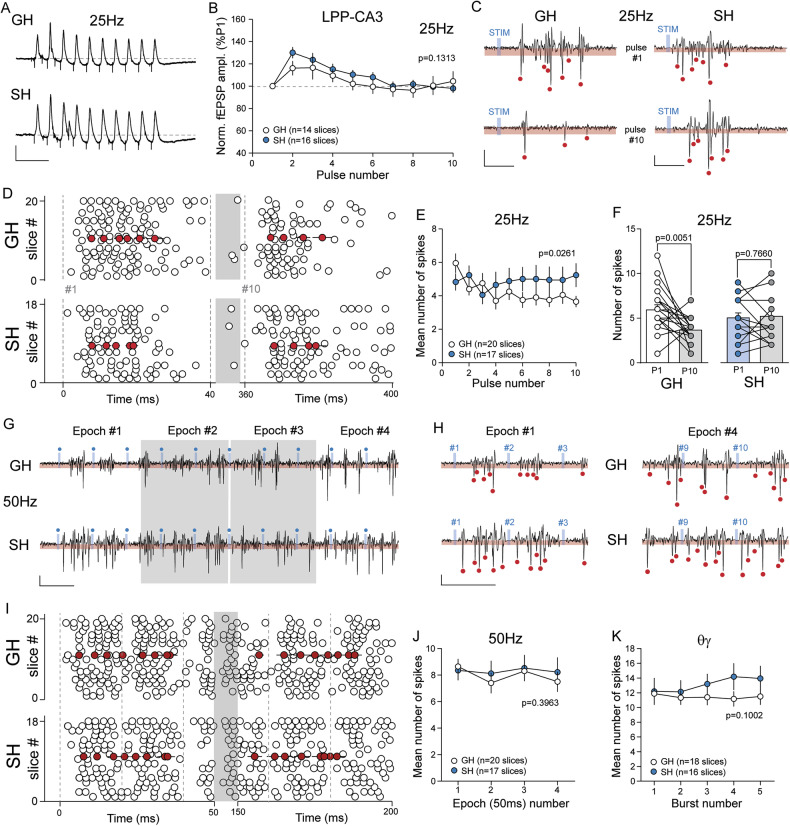
Frequency-dependent CA3 operations are selectively impaired following single housing. (**A)** Representative traces recorded from the CA3 pyramidal cell layer in hippocampal slices derived from GH (*top*) and SH (*bottom*) mice following 25 Hz (10 pulses) LPP stimulation (scale bars: y = 0.5 mV, x = 50 ms). (**B**) Graph summarizing the within train facilitation of the fEPSP amplitude recorded from GH and SH slices in response to brief (10 pulses) 25 Hz LPP stimulation. (**C**) Exemplar filtered LPP-evoked CA3 responses to the 1^st^ (*top*) and 10^th^ (*bottom*) pulse of a 25 Hz train (STIM, stimulation) recorded from a GH (*left*) and SH (*right*) slice (scale bars: y = 100 μV, x = 10 ms). Single units are identified (red dots) for each. (**D**) Raster plots illustrate the distribution of LPP-evoked CA3 spike times during the 1^st^ (*left*) and 10^th^ (*right*) response of a 25 Hz train for each GH (*top*) and SH (*bottom*) slice. In both cases, open circles indicate the time of individual spikes for each slice (i.e., y axis) while red circles show the mean ( ± SEM) spike times. (**E**) Graph summarizing the mean number of LPP-evoked CA3 spikes in response to each pulse of a 25 Hz (10 pulse) train in GH and SH slices. (**F**) Bar graphs show the number of CA3 spikes elicited on the 1^st^ and 10^th^ stimulation pulse of a 25 Hz train in slices derived from GH (*left*) and SH (*right*) mice. (**G,H**) Filtered LPP-evoked CA3 responses to a brief (10 pulse; blue dots) 50 Hz stimulation train recorded from representative GH (*top*) and SH (*bottom*) slices (**G**). In **H**, responses to the 1^st^ and 4^th^ 50 ms epochs for each are illustrated on an expanded timescale (scale bars for both: y = 100 μV; x = 20 ms); single units are indicated by red dots. (**I**) Raster plots show the distribution of LPP-evoked CA3 spiking during the 1^st^ and 4^th^ epoch (50 ms) of a 50 Hz train for each GH (*top*) and SH (*bottom*) slice. Open circles indicate the time of individual spikes while red circles show the mean ( ± SEM) spike times. (**J,K**) Graphs summarizing the mean number of CA3 spikes elicited in (**J**) each 50 ms epoch across the 50 Hz stimulation train and (**K**) each burst of a theta-gamma stimulation train (5 bursts) in GH and SH slices. For panels B, E, J and K, p-values are for GH vs SH; see Supplementary Table 1 for statistical details.

In contrast to single-pulse LPP activation, marked group differences emerged with beta frequency (25 Hz, 10 pulses) stimulation. The LPP-evoked fEPSP generated by the CA3 recurrent system (Supplementary Fig. 5A) expressed a modest frequency facilitation during 25 Hz stimulation that was unaffected by single housing (Fig. 5A, B & Supplementary Fig. 5B). However, in slices from GH mice the number of EPSP-associated spikes declined across the 25 Hz train, an effect that was absent in the SH group (Fig. 5C-F & Supplementary Fig. 5C). These findings help explain why CA1 output to beta frequency LPP activation is suppressed in GH but not SH mice. Results for gamma stimulation of the LPP were surprising: Despite strong filtering by LPP-DG terminals (Supplementary Fig. 3A-D), the CA3 spike output was remarkably constant across successive stimulation pulses in both groups (Fig. 5G-J & Supplementary Fig. 5D). When stimulation was delivered as theta-gamma bursts, the CA3 spike output was again consistent across successive bursts in both groups (Fig. 5K). This suggests that a step-down performed by LPP-DG during gamma is offset by amplification within CA3 and that these operations are unaffected by the single housing induced, depression-like state. It follows then that the gamma processing defect recorded in CA1 produced by the single housing protocol is located at some point downstream from CA3. Of note, the CA3 response to LPP stimulation at 5 Hz was, as anticipated based on the CA1 response, virtually identical between groups (Supplementary Fig. 5E, F).

### CA3 interneuron recruitment is required for filtering of beta frequency LPP activation

Spike responses to within-train facilitation are believed to be regulated by feedforward and/or feedback inhibition that shunts excitatory current [75]. Thus, perturbations in GABA_A_R expression following single housing could explain the impaired CA3 beta filter in SH mice. However, we found no differences in the number of synapse-sized, gephyrin-positive elements in CA3b str. radiatum, the lamina innervated by the recurrent collateral system, between groups (GH: 27,467 ± 1814 (mean ± s.d.), SH: 27,591 ± 1859, *P* = 0.9179; counts per 18,560 µm^3^). Furthermore, FDT analysis revealed that immunolabeling densities for the γ2 subunit, which is expressed in the majority of synaptic GABA_A_R isoforms [76], and the α2 subunit, one of the primary α subunits expressed by hippocampal pyramidal cells [77, 78], were virtually identical for GH and SH groups (Supplemental Fig. 5G-I). These data indicate that the deficit in the CA3 beta filter following SH is not due to sizeable alterations in the expression of postsynaptic GABA_A_Rs.

**Fig. 6:**
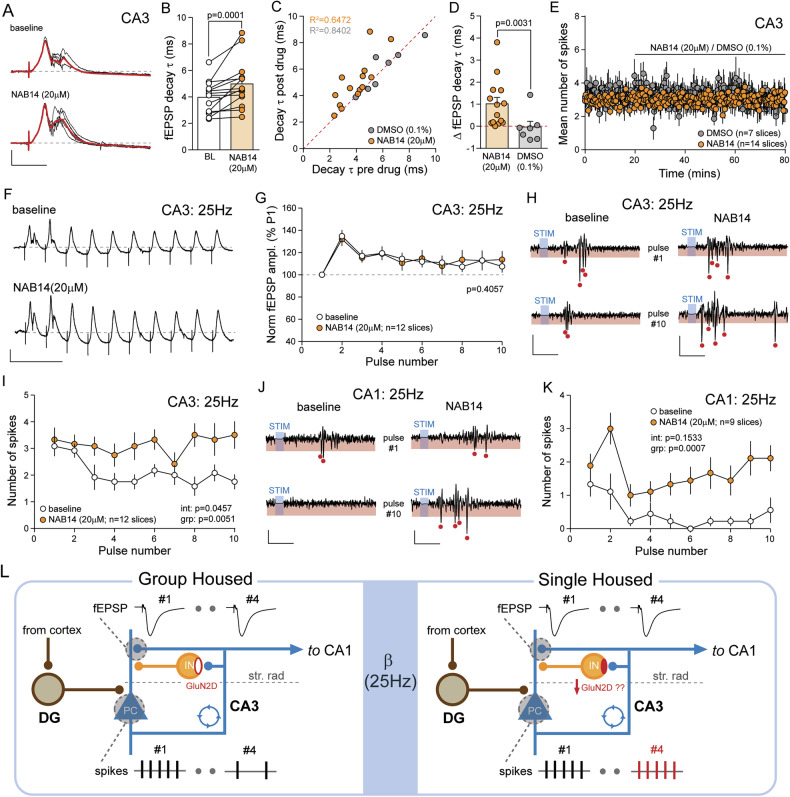
Recruitment of CA3 interneurons mediates the beta filter within the hippocampal circuit. (**A**) Representative LPP-evoked fEPSPs recorded from CA3 str. pyramidale prior to (*top*) and following (*bottom*) treatment with NAB14. The ensemble average fEPSP for each is shown in red (scale bars: y = 0.5 mV, x = 20 ms). (**B**) Graph showing the decay τ of LPP-evoked fEPSPs recorded from CA3 at baseline (BL) and following bath application of NAB14. (**C**) Scatter plot with unity line (red dashed) showing the relationship between the decay τ of LPP-evoked CA3 responses prior to and following treatment with NAB14 or vehicle (DMSO). (**D**) Graph shows the change in the fEPSP decay τ following NAB14 and DMSO treatment. (**E**) Graph showing the mean number of LPP-evoked CA3 spikes prior to, and during 60-min bath application of NAB14 or DMSO. (**F**) Representative traces recorded from the CA3 pyramidal cell layer of hippocampal slices following 25 Hz (10 pulses) LPP stimulation prior to (*top*) and after (*bottom*) NAB14 treatment (scale bars: y = 0.5 mV, x = 100 ms). (**G**) Graph summarizing the within train facilitation of the fEPSP amplitude (ampl.) recorded in response to brief (10 pulses) 25 Hz LPP stimulation prior to (baseline) and following NAB14 application; data are normalized to the 1^st^ pulse (P1). (**H**) Exemplar filtered LPP-evoked CA3 responses to the 1^st^ (*top*) and 10^th^ (*bottom*) pulse of a 25 Hz train (STIM, stimulation) recorded prior to (*left*) and following (*right*) NAB14 application (scale bars: y = 100 μV, x = 10 ms). Single units are identified (red dots) for each. (**I**) Graph summarizing the mean number of LPP-evoked CA3 spikes in response to each pulse of a 25 Hz (10 pulse) train before and after treatment with NAB14. (**J**). Exemplar filtered LPP-evoked CA1 responses to the 1^st^ (*top*) and 10^th^ (*bottom*) pulse of a 25 Hz train recorded prior to (*left*) and following (*right*) bath application of NAB14, with single units identified (red dots) (scale bars: y = 50 μV, x = 10 ms). (K) Graph summarizing the mean number of LPP-evoked CA1 spikes in response to each pulse of a 25 Hz (10 pulse) train before and after NAB14 treatment. (**L**) Schematic illustrating responses elicited by 25 Hz LPP activation in slices derived from GH and SH mice. In control, GH animals (*left*), CA3 synaptic responses (fEPSPs) to beta (25 Hz) frequency LPP stimulation do not decrease during the train, yet pyramidal cell (PC) spiking is significantly attenuated -- pulse #1 elicits a string of spikes while pulse #4 produces a much weaker response. This CA3 beta filter requires recruitment of inhibitory interneurons (IN) via activation of GluN2D-containing NMDA receptors (open red oval). In SH mice (*right*), CA3 pyramidal cell spiking does not decrease during a beta train (pulse #4; red). Since no changes to the synaptic responses (fEPSPs) were detected, we suggest that the failure point is likely at the connection between the interneurons and their excitatory input (filled red oval). Abbreviations for L: DG, dentate gyrus; str. rad, stratum radiatum. For **K,** **I**: int, interaction effect; grp, group effect. See Supplementary Table 1 for statistical details.

An inability to initiate interneuron spiking provides an alternative explanation. Hippocampal interneurons selectively express GluN2D-containing NMDA receptors (NMDAR) [79]. This receptor isoform has reduced voltage-dependence, high agonist affinity (i.e., glutamate, glycine) and slow decay kinetics [80] making it ideally suited to modulate interneuron spike initiation, and hence the recruitment, of inhibitory circuits. To test this, we used the negative allosteric modulator of GluN2C/D-containing NMDARs, NAB14 [81]: Note, GluN2C-NMDA receptors are largely non-neuronal in hippocampus [82]. Consistent with a role for local GABAergic inhibition in regulating fEPSP duration, bath application of NAB14 produced modest, yet significant effects on the decay τ of single pulse LPP-evoked CA3 responses (Fig. 6A-D), while changes in the waveform amplitude and area did not differ from vehicle treatment (Supplemental Fig. 6A-E). The number of CA3 spikes elicited by single pulse LPP activation was unaffected by NAB14 treatment (Fig. 6E & Supplemental Fig. 6F-I). However, LPP activation with brief beta frequency (10 pulses, 25 Hz) stimulation revealed a significant role for inhibitory circuits in the filtering of these inputs across the circuit. While facilitation of CA3 fEPSPs elicited by beta frequency activation was unaffected by NAB14, the within train suppression of cell spiking was completely attenuated (Fig. 6F-I). Such effects upon CA3 spiking appear, at least in part, to affect the broader circuit operation, as the typical filtering of CA1 spiking in response to 25 Hz LPP activation was absent with NAB14 treatment (Fig. 6J, K & Supplemental Fig. 7A). As expected, the single pulse LPP-CA1 spike output was, apart from first spike latency, unaffected by NAB14 (Supplemental Fig. 7A-E). These data indicate that recruitment of CA3 interneurons, via activation of GluN2D-containing NMDARs, is critical for the filtering of beta frequency inputs across the hippocampal circuit and inhibition of these receptors promotes circuit deficits reminiscent of single housing.

## Discussion

Although disturbances to the hippocampus are closely associated with the onset and progression of MDD [12], the impact that such changes have upon signal processing across the hippocampal circuit is largely unknown. Here, we tested if a depression-like condition in mice, characterized with behavioral and neurochemical measures, produces alterations in hippocampal circuit function and if such changes reflect generalized vs. discrete impairments. To address this, we used newly introduced techniques for sampling throughput across the primary hippocampal circuit, from cortical input to CA1 spike output [51]. This approach is agnostic with regard to the nature or site(s) of potential defects but instead assumes that even small disturbances may significantly affect circuit-level processing of patterned input. If so, subsequent examination of links and nodes within the circuit should reveal the nature and location of the disturbances generated by the experimental treatment. Because the assay is conducted using slices, any deviations from normality can be assigned to stable changes within the circuit rather than being reflections of behavioral abnormalities. We report that although the complex CA1 response to single pulse LPP activation was unaffected by single housing, sizeable signal processing defects emerged during patterned activation of the circuit. Further analyses showed that the depressed state was associated with discrete rather than global changes to the network.

Regarding the model, we found that a brief period of single housing (7-10 days) in a familiar environment and without added stressors produced multiple, diverse signs of depression in mice that were similar to those reported following more prolonged (> 2 weeks) isolation [37, 39]. The animals were less social, despite spending a seemingly normal amount of time investigating the environment, and showed a significant behavioral signature on two tests commonly used to screen antidepressant drugs. The SH mice also failed to acquire information about the identity and sequence of multiple cues, two operations that are fundamental to the formation of episodic memory. Impairments to episodic memory are a well-documented concomitant of depression [83–85] and one with particular importance for the cognitive problems that accompany the disorder [86, 87].

In addition to this behavioral phenotype, single housing induced strong activation of the LHb. The present findings are consistent with the effect of 24 h single housing [88], but also indicate that altered habenular activity is maintained over longer periods of isolation. Similar increases in Fos expression has been observed in other mild chronic stress models, presumably reflecting increased activity of LHb neurons [68]. Considerable data exists linking LHb activation to behavioral depression, and researchers have proposed an LHb hyperactivity hypothesis for MDD [65–68, 89, 90]. The robust habenular response to single housing further supports the conclusion that the 7-10 day protocol used here produces a depression-like condition in mice. Whether the elevated activity seen in SH mice is linked to the observed defects in hippocampal circuit operations is uncertain. The ventral striatum receives inputs from ventral CA1 and the subiculum and, in turn, provides a major input to the LHb [91–94]. Moreover, lateral hypothalamic neurons that drive inhibitory cells within the LHb [95, 96]. Field CA1 has extensive connections across the anterior-posterior levels of the hypothalamus [97] and accordingly may target these LHb projecting neurons.

The recently described input-output relationship of the hippocampus [51] provides a robust set of endpoint measures against which one can assess the effects of single housing on hippocampal circuit operation(s) of this structure. Single-pulse LPP stimulation produces a two-part CA1 fEPSP generated by the direct (LPP-CA3) and indirect (LPP-DG-CA3) connections between the LPP and CA3, with the CA1 spiking response driven by the latter sub-circuit. Moreover, it is clear that the hippocampus does not operate as the conventional tri-synaptic circuit but rather requires the mobilization of the dense recurrent CA3 system by the indirect path (LPP-DG-CA3-CA3). The resultant stereotyped, prolonged CA3 spike output amplifies the incoming LPP signal(s) to drive throughput [51]. Such arrangements ensure a reliable CA1 response but introduce a temporal component (reflecting recruitment of recurrent activity) that underlies the surprisingly delayed ( ~ 20 ms) CA1 output.

Despite the considerable complexity of the above arrangements, single housing had minimal effects on CA3 or CA1 responses generated by single-pulse LPP activation. In contrast, clear differences between the experimental groups emerged when the circuit was engaged by physiologically relevant frequencies and patterns. The operational states initiated by such inputs are shaped by interactions between frequency-dependent processes such as vesicle release, dendritic integration, and interneuron activity within the hippocampal subfields. Although descriptions exist for a significant number of these operations in isolation, it has become apparent that their composite effects on circuit-level operations are difficult to predict from first principles [51]. Findings here replicated the basic observations that in control (GH) mice, the system operates as a low pass filter [51] and additionally revealed that the CA1 spike output elicited by beta frequency inputs is heavily filtered. This latter point was surprising because synapses at the excitatory links in the circuit exhibit frequency facilitation [74], indicating the presence of a critical step between excitatory input and spike initiation. The present results demonstrate that this suppression of action potential firing reflects the recruitment of inhibitory transmission in CA3. Single housing significantly attenuated the low pass filtering of inputs arriving at beta and gamma frequencies, while a marked amplification of spiking was evident across successive theta-gamma bursts.

The SH-induced impairment of the CA3 beta filter was not related to alterations in the complex, frequency-dependent operations at either LPP-DG or CA3-CA3 terminals. Furthermore, we found no evidence that broad deficits in postsynaptic GABA_A_R-mediated inhibition underlie reduced filtering of beta frequency inputs in CA3. Rather, we propose that the major defect responsible for the loss of CA3 filtering in the SH mice is located at the excitatory synapses between recurrent collaterals and interneurons (Fig. 6L). Inhibition of GluN2C/D-containing NMDARs, a subtype almost exclusively expressed on interneurons in hippocampus [79] produced a CA3 and CA1 spike response to 25 Hz LPP activation incredibly similar to that observed following single housing. The CA3 spike output did not depress during a 50 Hz, gamma train in either group, despite filtering at the upstream LPP-DG synapse. This result points to the unexpected conclusion that gamma processing includes amplification in CA3 of a suppressed signal from the DG, an effect that may reflect frequency-dependent mobilization of local inhibitory cells [98]. Given that CA3 responses to gamma (as opposed to beta) LPP activation were not different between groups, we assume that the filtering defect shaping CA1 firing in SH mice occurs at some point beyond CA3. Indeed, treatment with NAB14 significantly reduced the CA1 first spike latency elicited by single pulse LPP activation, with no apparent effects upon the CA3 spike output. The mobilization of inhibitory interneuron subtypes in CA2 and/ or CA1, presumably via activation of GluN2D-containing NMDARs, provides a potential mechanism for such effects on signal throughput.

Whether these frequency-dependent operations are mediated by a specific interneuronal subtype(s) residing at distinct locations within the circuit remains to be established. Despite detailed descriptions of the anatomical, neurochemical and functional properties of interneuron subtypes in hippocampus [99–101], their respective role(s) in shaping throughput of cortically-evoked signals are largely lacking. Our results indicate that the recruitment of CA3 interneurons contributes to the filtering of beta frequency inputs. Unlike CA1, within CA3 a significant number of somatostatin (SST) interneurons are located in strata pyramidale, radiatum, and lucidium, with their terminal zone proximal to the pyramidal cell (PC) body layer [101]. This, in conjunction with their ability (albeit in CA1) to follow high frequency inputs [102], suggests they may play a more prominent role in modulating PC spiking. Given that cortico-limbic SST levels and SST-expressing cell counts are reduced in depressed individuals and preclinical rodent models [103], it seems plausible that these cells may be important in the suppression of CA3 spiking during beta frequency input as well as the development and/ or progression of depressive disorders. The responses to gamma frequency LPP activation appear more complex. Given the robust CA3 spiking elicited by 50 Hz LPP activation, it is likely that this signal is filtered at some point between CA3 and CA1. Parvalbumin (PV)-expressing interneurons in CA1 make an attractive candidate for such a role. These cells can elicit high frequency spike output (i.e., >150 Hz) and receive highly efficacious glutamatergic input: high probability release (*p*) terminals (from CA3/ CA1 PCs) and rapidly gated AMPA receptors [104, 105]. Given the observation that different CA3 PCs are engaged across successive pulses [51], one would anticipate PV cells to be reliably recruited, and to suppress CA1 PC output by shortening the window for spike initiation [106, 107]. Finally, one should not overlook the functional significance of the two distinct serial inhibitory circuits in CA3 (i.e., the mossy fiber-interneuron and recurrent associational PC-interneuron systems). Both circuits display striking differences in frequency tuning that likely contribute to CA3 spike output elicited by gamma frequency activation [98] (Gunn, Lynch unpublished observation).

The disturbances to signal processing by the circuit likely contribute to certain signs of psychological depression exhibited by the SH mice. The network provides dense feedback to association neocortex, the route generally assumed to promote acquisition and retrieval of episodic memories. Impaired filtering could represent a central factor in the episodic memory problems found in the mice. There is also the possibility that malfunctions in the basic hippocampal circuit contribute to the affective components of depression through the structure’s dense projections to subcortical areas that are related to arousal, anxiety, and mood. The best known of these involves a series of links running from the subiculum, the primary target of CA1, to the mammillary bodies and anterior thalamus and then to the anterior cingulate cortex (the ‘Papez circuit’) [108]: the latter has long been associated with MDD [109–112]. As discussed above, a second set of descending hippocampal/subicular projections innervates a column of basal forebrain structures that access dorsal (stria medullaris) and ventral (medial forebrain bundle) pathways to the midbrain monoamine systems [113–115]. The habenula is the primary station along the dorsal pathway and, as noted, is intensely activated in SH mice during the three chamber social task. It remains for future work to determine if i) manipulations of the hippocampal circuit produce signs of depression and ii) antidepressant treatment can normalize circuit function and behavior in SH mice.

Collectively, these data lead to the simple and testable hypothesis that alterations in recruitment of hippocampal interneurons underlie the initiation and/or progression of depression. In this regard, perturbations in GluN2D-containing NMDARs on these inhibitory cells (e.g., levels, composition) provide an attractive mechanism. Indeed, recent studies have postulated that GluN2D-NMDARs are targeted by the antidepressant ketamine [116]. Given that MDD is significantly more prevalent in females [117, 118], establishing whether the behavioral and circuit deficits induced by single housing in male mice are similar or entirely different in females is a key question to address. Based on the preclinical and clinical observation that females outperform males in specific aspects of episodic memory [54, 119], it is possible that there are sex differences in hippocampal circuit function for females which may be especially vulnerable to depression.

## Supplementary information

Supplemental Material

## Data Availability

Data and code for electrophysiological analysis are available upon reasonable request.
