# Supplementary material for: A depression–like phenotype is associated with discrete defects in the primary hippocampal circuit
Source: Transl Psychiatry. 2026 May 16;16:350. doi: 10.1038/s41398-026-04094-3 (PMC13346759; doi:10.1038/s41398-026-04094-3)
Supplement: Supplementary file 1 — Supplemental Material [file 41398_2026_4094_MOESM1_ESM.pdf]

SUPPLEMENTAL MATERIALS

Supplemental Methods

Animals

Studies used young adult (2-3 mo old) C57BL/6 male mice. Mice were group-housed with littermates (3-5 per cage), or single housed (7-10 days) in vivarium at 68°F and 55% humidity with a 12 hr on/12 hr off light cycle and lights on at 6:30AM; food and water were provided *ad libitum*. Behavioral experiments were always performed between 9AM – 12PM. Experimental mice were not prior handled. Experiments were conducted in accordance with the National Institutes of Health Guide for the Care and Use for Laboratory Animals and protocols approved by the Institutional Animal Care and Use Committee at the University of California, Irvine.

Behavioral Assays

All behavioral tasks were video recorded digitally using an overhead or lateral positioned camera (Logitech C920x HD Pro Webcam, 1080p/30fps). Each video was scored by two experimenters blind to group and the average of the two scores were used.

Three-Chamber Task

For this task we used a modified version of the widely accepted sociability paradigm developed by Crawley and colleagues.[1] Prior to the task, conspecific animals were habituated to an inverted wire cup (10cm diameter) for 10 min three times, with 10-minute breaks in between. On the day of experimentation, the test animal was habituated (10 min) in a large Plexiglass arena (60 cm × 40 cm × 20 cm) with an open top and 3 equal-sized chambers separated by 2 transparent walls with doorways. After habituation, the animal was placed back in the center

chamber with interior doors closed. Social Approach Phase: The animal was allowed to re-explore the chambers for 10 min, with an empty inverted wire cup in one side chamber (C1), and inverted wire cup containing a conspecific mouse in the other side chamber (C2). Time spent exploring the conspecific male mouse vs. the empty cup was collected. The animal was then returned to the center chamber. Social Recognition Phase: The animal was given 10 min to explore the chambers containing the familiar mouse that was now positioned in C1 vs. a novel mouse positioned in C2. Time spent exploring both cups was collected: exploration time was scored when the animal oriented its nose towards the cup and was within ~0.5 cm of its proximity. Gnawing or climbing over the cups was not included. A discrimination index (DI) was calculated for the social approach and social recognition as follows: 100 x $(t_{novel/\ mouse} - t_{familiar/\ object})/(t_{total\ sampling})$. Finally, a subset of mice that were tested in the three-chamber task were euthanized 90 min following the completion of the task and brains were processed for Fos immunoreactivity as described below.

Novel Object Recognition Test

Animals were placed into a Plexiglass arena (30 cm × 25 cm x 21.5 cm) and allowed to explore freely for 5 min. Then, 2 identical round objects (base diameter of 6 cm) were placed in the arena for animals to explore for another 5 min. Animals were placed back in their home cage and returned to vivarium for 24h. For testing, animals were placed back into the same arena, with one of the round objects replaced with a novel square object (5cm x 5cm). Animals were allowed to explore for 5 min. Time spent exploring the familiar and novel objects were collected: exploration time was scored when the animals nose sniffed the objects within ~0.5 cm. Percent novelty preference was calculated as 100 x $[t_{novel}/(t_{novel} + t_{familiar})]$.

Tail Suspension Test

This test was conducted as described.[2] Using a 17 cm strip of tape (1 cm wide), 2cm was applied to the end of each animal's tail, with 2-3 mm of the tail tip remaining outside of the tape. The remaining free length of the tape (15 cm) was used to hang the animal from an overhead platform. A cone (2cm length) was wrapped at the base of the animal's tail to prevent it from climbing onto their tail during testing. Animals were suspended 45cm above a soft platform for 6 min. After testing, the animal was removed from the platform and returned to their home cage. An Immobility score for each animal was calculated based on the period (seconds) during which the animal's hind legs were immobile during the 6 min.

Forced Swim Test

Animals were placed in a Plexiglas cylinder water tank with water temperature at 24-25°C, as measured using a thermometer (Easy-Read® thermometer, HB Instruments B60304-0500). The water level was 15 cm above the bottom of the tank. Animals were recorded for 6 min in the water tank, with scoring of mobility (seconds) for the last 4 min of the task. Mobility was categorized as their hind legs moving, or any movements necessary to keep their head above water. An immobility score was calculated as [240 seconds – seconds of mobility]. After the task, animals were dried with Kimwipes and returned to their home cage with a heating pad underneath for 1 hr to prevent hypothermia.

Palatable Food Consumption

Mice were given palatable food (cocoa pebbles cereal (Post, USA; ~1g/mouse) for five consecutive days in a foreign cage and allowed to freely consume for 1 hr. Food was weighed prior to each feeding session. At the end of the procedure on day 5, the remaining food was removed and weighed to measure amount of consumption.

Episodic Memory 'What' Task

To assess encoding of cue identify ('what') information, a serial odor task was used in which greater time sampling a novel odor D vs. a previously presented odor A at testing is interpreted as evidence that the animal had acquired the identity of the familiar cue.[3, 4] Odors were diluted in mineral oil and 120 µl of the scented mixture was pipetted onto filter paper (final concentration of 0.1 Pascals) which was placed in a glass jar (5.25 cm diameter x 5 cm height) with a plastic lid containing a small (~1.5 cm diameter) hole to allow the mouse to explore the odorant. During the habituation session, two jars (without odor) were placed at each end of a plexiglass box (30 cm x 25 cm floors with 21.5 cm walls), and the mouse was allowed to explore for 5 min. The animal was removed to a holding cage and the jars were changed for the next step. The mouse was then presented with a series of odorant pairs (A:A>B:B>C:C) for 5 min each and 3 min between pairs; the animal was moved to the holding cage between odor pairs. In the final test session, the mouse was exposed to the odorant pair A:D and allowed to explore for 5 min. A mouse was scored as exploring an odor whenever their nose was within 0.5 cm of, and directed towards, the odor hole. A discrimination index (DI) was calculated for as follows: 100 x (t_{novel} – $t_{familiar}$)/($t_{total\ sampling}$).

Episodic Memory 'When' Task

This paradigm was largely identical to the 'what' task, except that an additional odor pair was added to the last step in the exposure sequence (i.e. odor D).[3, 4] After exposure to odor pair D, the mouse is presented two different odors selected from the earlier odor pairs (B and C). In general, group housed control mice explore the odor seen least recently (B) vs. and odor explored more recently (C). Preferential exploration of the cue encountered earlier from a cue presented later in that series indicates that the animal distinguishes the temporal order of cues sampled within an odor series. DI's were calculated as follows: 100 × (t_{novel} – $t_{familiar}$)/($t_{total\ sampling}$).

For all memory tests, animals were verified for exploration of each odor/ object for at least 1 second during exposure and testing, and location of test odors were counterbalanced.

Odorants for behavioral tasks

Odorants used were as follows: odor A: (+)-Limonene (≥97% purity, Sigma-Aldrich); odor B: Cyclohexyl Ethyl Acetate (≥97%, International Flavors & Fragances Inc.); odor C: (+)-Citronellal (~96%, Alfa Aesar); odor D: Octyl Aldehyde (~99%, Acros Organics).

Episodic Object Recognition Test

Animals were placed into a Plexiglass arena (30 cm × 30 cm x 21.5 cm) and allowed to explore freely for 5 min. Then, 4 distinct objects (rugged cone with 5 cm base diameter, cone with 4.5 cm base diameter, 3x3x3cm triangle, and 3x3cm square) were placed in the arena for animals to explore for another 5 min. Animals were placed back in their home cage and returned to vivarium. 24hrs later, animals were placed back into the arena with the same objects except the square was changed out for a novel circle object (4 cm diameter). Animals were allowed to explore for 5 min. Time spent exploring the familiar and novel objects were collected. All animals were verified for exploration of all objects for at least 1second in both the exposure and testing trials. DI's were calculated as: $100 \times (t_{novel} - t_{familiar})/(t_{total\ sampling})$.

Immunofluorescence and microscopy

Fos expression

A subset of mice that had undergone the three-chamber task were assessed for neuronal activity in the habenula using the cell activity marker Fos.5 Ninety minutes after completion of the task, animals were perfused with 4% paraformaldehyde in 0.1M phosphate buffer (pH 7.2, PB), postfixed for 2 hr, and then cryoprotected overnight in 20% sucrose in 0.1M PB at 4°C. Frozen tissue sections (30 um thick) were collected through the rostrocaudal extent of the

habenula and processed for Fos immunofluorescence using rabbit anti-Fos (Novus, NBP2-50057SS; 1:5K) and Alexa Fluor488 anti-rabbit secondary. Briefly, sections were incubated in primary antisera for 24h, rinsed in 0.1M PB and incubated in secondary antisera for 2h at RT; all antibody incubations were in 0.1M PB containing 0.3% Tri-X and 3% normal swine serum. Following washes in 0.1M PB, tissue was coverslipped using VectaShield with DAPI (Vector labs). Photomicrographs of Fos immunolabeling in 8 spaced coronal sections through habenula were collected at 10x magnification using a Leica DMI6000B epifluorescence microscope with an sCMOS pco.edge camera and MetaMorph v7.8.7.0 software. Fos-labeled cells were quantified (cells/area) in defined/outlined regions of interest using ImageJ by two individuals blind to groups. Automated techniques were used to count the number of labeled cells in the medial and lateral habenula. Images were first thresholded using MaxEntropy and then cells were counted using the Analyze Particles function with 100-infinity pixel^2 size and 0.3-1.0 circularity. Counts were verified by hand for each image. Cell counts were collected across the rostrocaudaul extent of the habenula and then averaged per animal.

$GABA_A$R subunit levels at inhibitory synapses

Mice were perfused with 4% paraformaldehyde in 0.1M phosphate buffer (pH 7.2) and brains processed for dual immunofluorescence as previously described.[6] Sections were incubated in primary antisera at 4°C for 24hrs, followed by 2hr incubation in secondary antisera at RT. Following a final wash in 0.1M PB, tissue was cover slipped using VectaShield with DAPI. Primary antisera cocktails contained the following antibodies for the scaffold protein gephyrin and specific $GABA_A$R subunits (i.e., (α2 and γ2): guinea pig anti-gephyrin (1:1000; Synaptic Systems,147318) combined with either rabbit anti-α2 (1:3000; Synaptic Systems 224103) or rabbit anti-γ2 (1:1000 Synaptic Systems, 224003). Buffers were the same as for Fos experiments.

Fluorescence deconvolution tomography (FDT) was used to quantify densities of $GABA_AR$ subunits at gephyrin-positive synapses as described.[4, 6, 7] Briefly, image z-stacks from str. radiatum of CA3b were collected at 63x (1.4NA) using a Leica DM6000B epifluorescence microscope equipped with a Ludl stage with a BioPreceision Stepper Motor driven by Velocity 4.0 software (PerkinElmer, Waltham, MA, USA); Z-stacks were collected at intervals of 0.2 µm through a depth of 2µm in a sample field measuring 135 x 105 x 2um (28,350um^3). For each brain, five tissue sections were sampled. Quantitative FDT analysis entailed processing images through restorative deconvolution (99% confidence; Volocity 4.0, PerkinElmer) and then analyzing 3-dimensional (3-D) constructed montages for synaptic labeling using in-house software (c99, Java (OpenJDK IcedTea 6.1.12.6), Matlab R2019b, PuTTY 0.74, and Perl 5.30.0). Within each montage, immunolabelled objects were detected using threshold segmentation separately for each channel: the image was normalized for background density and objects were segmented based on connected pixels above threshold. Immunofluorescent elements meeting the size and eccentricity constraints of synapses, and detected across multiple intensity thresholds, were quantified using automated systems. Elements were considered double-labelled if there was contact or overlap of fields occupied by the two fluorophores as assessed in 3-D. For each z-stack of field CA3 these procedures identified approximately 20-30 thousand reconstructed terminal synapses. Based on the maximum fluorescence intensity of each image, counts of double-labeled (2x) puncta were assigned to ascending density bins and the data were plotted as intensity frequency histograms. Labeled puncta with immunofluorescence density at ≥110 on the arbitrary 255 step scale were considered densely-labeled. Counts of densely-labeled puncta for 5 sections per animal were averaged to generate mean animal values. Graphs show group mean ± SEM values.

Electrophysiological Recordings

Hippocampal slices were prepared as previously described.[8] Experiments were initiated from 8-10AM. Brains were rapidly removed from the cranium and placed in ice cold, oxygenated (95% O_2/ 5% CO_2) high Mg^{2+}, artificial cerebrospinal fluid (HM-aCSF) containing (in mM): 87 NaCl, 26 $NaHCO_3$, 25 glucose, 75 sucrose, 2.5 KCl, 1.25 NaH_2PO_4, 0.5 $CaCl_2$, 7 $MgCl_2$, (320-335 mOsm). Horizontal sections (400μm) were cut using a Leica Vibrotome (model VT1000s, Deer Park, IL, USA) in ice cold (4°C) HM-aCSF and rapidly transferred to an interface recording chamber containing a constant perfusion (60-70 ml/hr) of oxygenated (95% O_2/ 5% CO_2) aCSF containing (in mM): 124 NaCl, 3 KCl, 1.25 KH_2PO_4, 1.5 $MgSO_4$, 26 $NaHCO_3$, 2.5 $CaCl_2$, and 10 glucose (300-310 mOsm, pH 7.4, 31±1°C). Recordings began 1-1.5hrs later. All extracellular recordings were digitized at 20 kHz using an AC amplifier (A-M Systems, Model 1700) and collected using NacGather 2.0 (Theta burst Corp.).

Signal throughput CA1*:* To analyze signal throughput, 400μm slices were prepared using the temporal half of the hippocampus. This typically yielded four slices (for each hemisphere) where optimal responses were generally obtained from the middle two slices. A stimulating electrode (twisted nichrome wire) was placed in the dentate gyrus (DG) outer molecular layer (OML) towards the apex of the two granule cell blades targeting the direct and indirect lateral perforant path (LPP) projections while two recording pipettes were positioned in CA1c stratum (str.) radiatum and str. pyramidale (see **Fig. 3A**). Single pulse stimulation produced a complex two part field excitatory postsynaptic potential (fEPSP) and stimulation intensity was set such that single units were reliably observed on the second component in the CA1 pyramidal cell (PC) layer response. A baseline period (>20-min) of single pulse stimulation (0.05Hz) was recorded, with spontaneous activity (i.e., single units, sharp waves [SPW]) collected over the 10-second period following each stimulation pulse. To test the effect that repetitive LPP stimulation had upon CA1 spike output, brief trains were delivered across a range of frequencies (i.e., (θ [5Hz],

β [25Hz] and γ [50Hz]) or patterns (θγ; 5 bursts). Stimulation trains were delivered in a random order and separated by a minimum of 10 min. Stimulating electrode placement was confirmed by recording LPP-evoked fEPSPs in CA3 str. lacunosum and testing the response to paired pulse stimulation (40ms interval): Only slices displaying robust paired pulse facilitation, indicating that the activated fibers belong to the LPP rather than medial perforant path (MPP),[9] were included in the analysis.

LPP-CA3 responses*:* A stimulating electrode was positioned in the DG OML (as above and **Fig. 6A**) and a single recording pipette was positioned in the CA3a PC layer. A subset of experiments included a second recording pipette in CA3 str. radiatum. The stimulation intensity was set to evoke a modest fEPSP (0.5-1mV) that reliably contained single units. A baseline period (>20-min) of single pulse stimulation (0.05Hz) was recorded, with spontaneous activity (i.e., single units, SPWs) collected over the 10-seconds following each stimulation pulse. After a stable period of baseline responses (10-min), the effect of repetitive stimulation was assessed by delivering brief trains across a range of frequencies (i.e., 5Hz, 25Hz or 50Hz; 10 pulses) or patterns (theta-gamma; 5 bursts).

LPP-DG responses: A stimulating electrode was positioned in the outer third of the DG OML, while a recording pipette was placed either in the OML (dendritic response) or granule cell layer (population spike response). Paired pulse stimulation (40ms interval) was used to confirm the characteristic facilitation present at the LPP-DG synapse.[10, 11] To record dendritic responses, the stimulation intensity was adjusted to elicit a fEPSP that was ~50% of the maximum population spike free response, while for cell layer responses stimulation was adjusted to produce a population spike amplitude of 1-2mV: responses to 0.05Hz stimulation were recorded thereafter. In both cases, the effects of repetitive stimulation were tested after 10-min of stable baseline responses (at 0.05Hz) with brief 10 pulse trains delivered at 5Hz (θ), 25Hz (β) or 50Hz (γ). Stimulation trains were delivered in a random order and separated by at least 10-min.

Effect of NAB14 (20μM) upon signal throughput: A stimulating electrode was positioned in the outer third of the DG OML (as above) and recording electrodes placed in the pyramidal cell layer of CA3a and CA1c. The stimulation intensity was set such that i) the single units were reliably observed on the second component of the CA1 response and ii) a modest fEPSP (~0.5-1.5mV amplitude) that reliably contained spikes was elicited in CA3. An initial baseline period of single pulse responses (10-15 minutes; 0.05Hz) was recorded prior to delivering a single 25Hz (10 pulse) train. Following a second period of stable baseline responses (>20 minutes) the effect that bath application (60 minutes) of the GluN2C/D negative allosteric modulator (NAM) NAB14 (20μM) or vehicle (0.1% DMSO) had upon single pulse CA1 and CA3 responses. The effect that the NAM or vehicle treatment had upon the response to 25Hz (10 pulse) LPP activation was then determined after the 60 minutes treatment.

Analysis of data

All recordings were analyzed off line. The properties of the fEPSP waveform were analyzed using NacShow 2.0 (Theta Burst Corp), while spontaneous activity and evoked single units were analyzed using a custom code (Python version 3.8).

fEPSP analysis*:* LPP-evoked fEPSPs recorded from the apical dendrites (i.e., str. radiatum) of CA1c were analyzed with regard to the initial slope (20-80%) of the rising phase while responses recorded from the PC layer of the same subfield were analyzed with regard to peak amplitude. The coefficient of variation (CV; i.e., [SD/mean]*100) was calculated for each. Measurements were made for the initial and secondary components of the waveform. The LPP-DG dendritic responses elicited during the different stimulation trains (i.e., 5Hz, 25Hz, 50Hz) were analyzed with regard to peak amplitude. The amplitude of population spike recorded from the granule cell layer was measured for each response in the train. In both cases, responses were then normalized as the percentage ratio of the fEPSP amplitude or population spike

amplitude of the first pulse. LPP-evoked CA3 fEPSPs elicited during a 25Hz train were analyzed with regard to their peak amplitude and similarly normalized as the percentage ratio of the first pulse.

Evoked single units: LPP-evoked spikes recorded from CA1 or CA3 were analyzed using a custom-written computer code created with Python 3.8. Briefly, extracellular recordings (10 second sweeps) were fed through a band pass filter (300-5000Hz). Spike detection used a combined amplitude threshold (-45µV) and rate of rise threshold (240 µV/ms), with the spike output generated by single-pulse LPP activation analyzed across the 50ms period following the stimulation artifact. For each slice, responses to single pulse stimulation (30 consecutive) were analyzed for number of spikes, inter-spike interval (ISI), latency to 1st spike, spike amplitude and instantaneous frequency (i.e., 1/ISI) of spike output. The standard deviation of the 1st spike latency was used to describe the "jitter" within each slice.

Analysis of repetitive stimulation: The same custom code and detection parameters described above was used to analyze the CA1 and CA3 spike output to brief repetitive LPP stimulation at different frequencies (i.e., 5Hz, 25Hz, 50Hz) and patterns (i.e., theta gamma). LPP-evoked spikes were analyzed for each pulse with regard to number of spikes, latency to 1st spike and associated "jitter', ISI and instantaneous frequency of spike output. With 5Hz stimulation, spiking was analyzed for 50ms after the stimulation artifact associated with each pulse, while 25Hz stimulation was truncated to 40ms (i.e., the inter-pulse interval). With 50Hz or theta-gamma burst stimulation, spiking was assessed i) across the 20ms interval between gamma frequency pulses and ii) over 50ms periods following the initial pulse in the train (i.e., divided into four consecutive 50ms epochs) or burst. Assessing LPP-evoked spiking over such 50ms periods enabled quantitative comparison with single-pulse output and with responses to 5Hz stimulation.

Raster plots were generated to show the times at which individual spikes occur following single pulse and repetitive LPP stimulation. For single pulse LPP stimulation the distribution of individual spikes were plotted across the 50ms period after stimulation for 15 trials from representative slices derived from GH and SH mice. Similar plots were made for the spike responses elicited during repetitive stimulation (5Hz, 25Hz, 50Hz and $\theta\gamma$) for each slice from the GH and SH groups. The distribution of spikes (across time) was shown over 50ms and 40ms periods for responses to 5Hz and 25Hz LPP stimulations respectively. The response to 50Hz stimulation was generated for 50ms epochs (as above), while spiking during $\theta\gamma$ stimulations was displayed over 60ms (i.e., three 20ms periods). The mean time at which spikes occurred were calculated within (i.e., single pulse responses) or across slices (i.e., repetitive stimulation). A mean value (± SEM) was only calculated for a given spike (i.e., 1st, 2nd, 3rd) if that spike number was elicited in 50% or more of trials or slices. Typically for repetitive stimulation responses were shown for the 1st and last response (i.e., pulse, burst or epoch) for a given stimulation train.

Effect of NAB14 upon single pulse and repetitive LPP activation

fEPSP analysis*:* LPP-evoked fEPSPs recorded from the pyramidal cell layer (i.e., str. pyramidale) of CA3a were analyzed with regard to peak amplitude and area throughout the recording. An ensemble average fEPSP was generated (20 events) and used to measure the decay τ of the waveform using the following monoexponential equation: $Y(t)=A*\exp(-1/t)$. Treatment effect was assessed by comparing the fEPSP properties (i.e., peak amplitude, area) measured during the baseline period (20 minutes; 60 events) with those obtained from the final 10 minutes (i.e., 30 events) of the infusion period. The ensemble average generated by the last 20 minutes of the baseline and infusion period was used to determine effects upon the decay τ. Events contaminated with spontaneous sharp waves or spurious noise, were excluded from the

analysis. Slices where the fEPSP amplitude changed by 15% or more across the 20 minute baseline period were excluded from the analysis.

Evoked single units: As described above, LPP-evoked spikes recorded from CA1 or CA3 were analyzed using a custom-written computer code created with Python 3.8. Spike detection used a combined amplitude threshold (-25 μV or -45μV) and rate of rise threshold (240 μV/ms). The spike output generated by single-pulse LPP activation was analyzed across the 50ms period following the stimulation artifact. For each slice, baseline and treatment (i.e., NAB14 or DMSO) responses to single pulse stimulation were analyzed for number of spikes, latency to 1st spike and instantaneous frequency (i.e., 1/ISI) of spike output. The standard deviation of the 1st spike latency was used to describe the "jitter" within each slice. Treatment effect was assessed by comparing the spike properties measured during the baseline period (20 minutes; 60 events) with those obtained from the final 10 minutes (i.e., 30 events) of the infusion period. Events contaminated with spontaneous sharp waves or spurious noise, were excluded from the analysis.

Analysis of repetitive stimulation: The same custom code and detection parameters described above was used to analyze the CA1 and CA3 spike output to brief repetitive LPP stimulation at 25Hz before and after vehicle or drug treatment. LPP-evoked spikes were analyzed for each pulse with regard to number of spikes, latency to 1st spike and associated "jitter', ISI and instantaneous frequency of spike output. Spiking was analyzed for 40ms (i.e., the inter-pulse interval) after each stimulation pulse. LPP-evoked CA3 fEPSPs elicited during a 25Hz train before and after treatment were analyzed with regard to their peak amplitude and responses were then normalized as the percentage ratio of the fEPSP amplitude of the first pulse.

Statistical Analysis

All results are presented as group means ± SEM. Statistical significance was determined using GraphPad Prism (v6.0). All data was tested for normality using visual inspection of the distribution (i.e., Q-Q plot), followed by the D'Agostino & Pearson omnibus normality test. A *p*-value of <0.05 was considered significant. For paired data (i.e., within slice or animal) the distribution of the difference between the two observations was assessed for normality. In cases where the group size was too small to determine the distribution, we assumed that it was not normal and used the appropriate non parametric statistical test. When using the two-way ANOVA, the residuals (i.e., difference between observed and predicted values) for each group were tested for normality. Statistical comparisons between groups that were normally distributed were made using the paired or unpaired Student's t-test. The Mann-Whitney or Wilcoxon matched pairs tests were used for statistical comparisons between those groups that were not normally distributed. Statistical comparisons between different circuit configurations were made using two-way ANOVA (repeated measure [RM]), unless otherwise stated. For all electrophysiology experiments, the group 'n' refers to the number of slices used for recordings, which were obtained from a minimum of three animals per group. A *p*-value of <0.05 was considered significant for the unpaired Student's t-test and two-way RM ANOVA, with a more stringent criterion for significance (p < 0.01) used for the Kolmogorov–Smirnov test. In behavioral studies, for plots of % novelty the chance level was considered 50%, while in plots of discrimination index (DI) the chance level would be 0 (on the y axis). Details of all statistical analyses are presented in **Supplementary Table 1**.

References

1. Moy SS, Nadler JJ, Perez A, Barbaro RP, Johns JM, Magnuson TR *et al.* Sociability and preference for social novelty in five inbred strains: an approach to assess autistic-like behavior in mice. *Genes Brain Behav* 2004; **3**(5)**:** 287-302.

2. Can A, Dao DT, Terrillion CE, Piantadosi SC, Bhat S, Gould TD. The tail suspension test. *J Vis Exp* 2012; (59)**:** e3769.

3. Cox BM, Cox CD, Gunn BG, Le AA, Inshishian VC, Gall CM *et al.* Acquisition of temporal order requires an intact CA3 commissural/associational (C/A) feedback system in mice. *Commun Biol* 2019; **2:** 251.

4. Le AA, Lauterborn JC, Jia Y, Cox CD, Lynch G, Gall CM. Metabotropic NMDAR Signaling Contributes to Sex Differences in Synaptic Plasticity and Episodic Memory. *J Neurosci* 2024; **44**(50).

5. Morgan JI, Curran T. Proto-oncogene transcription factors and epilepsy. *Trends Pharmacol Sci* 1991; **12**(9)**:** 343-349.

6. Lauterborn JC, Scaduto P, Cox CD, Schulmann A, Lynch G, Gall CM *et al.* Increased excitatory to inhibitory synaptic ratio in parietal cortex samples from individuals with Alzheimer's disease. *Nat Commun* 2021; **12**(1)**:** 2603.

7. Wang W, Le AA, Hou B, Lauterborn JC, Cox CD, Levin ER *et al.* Memory-Related Synaptic Plasticity Is Sexually Dimorphic in Rodent Hippocampus. *J Neurosci* 2018; **38**(37)**:** 7935-7951.

8. Gunn BG, Pruess BS, Gall CM, Lynch G. Input/Output Relationships for the Primary Hippocampal Circuit. *J Neurosci* 2025; **45**(2).

9. Berzhanskaya J, Urban NN, Barrionuevo G. Electrophysiological and pharmacological characterization of the direct perforant path input to hippocampal area CA3. *J Neurophysiol* 1998; **79**(4)**:** 2111-2118.

10. Christie BR, Abraham WC. Differential regulation of paired-pulse plasticity following LTP in the dentate gyrus. *Neuroreport* 1994; **5**(4)**:** 385-388.

11. Wang W, Trieu BH, Palmer LC, Jia Y, Pham DT, Jung KM *et al.* A Primary Cortical Input to Hippocampus Expresses a Pathway-Specific and Endocannabinoid-Dependent Form of Long-Term Potentiation. *Eneuro* 2016; **3**(4).

Supplemental Figures

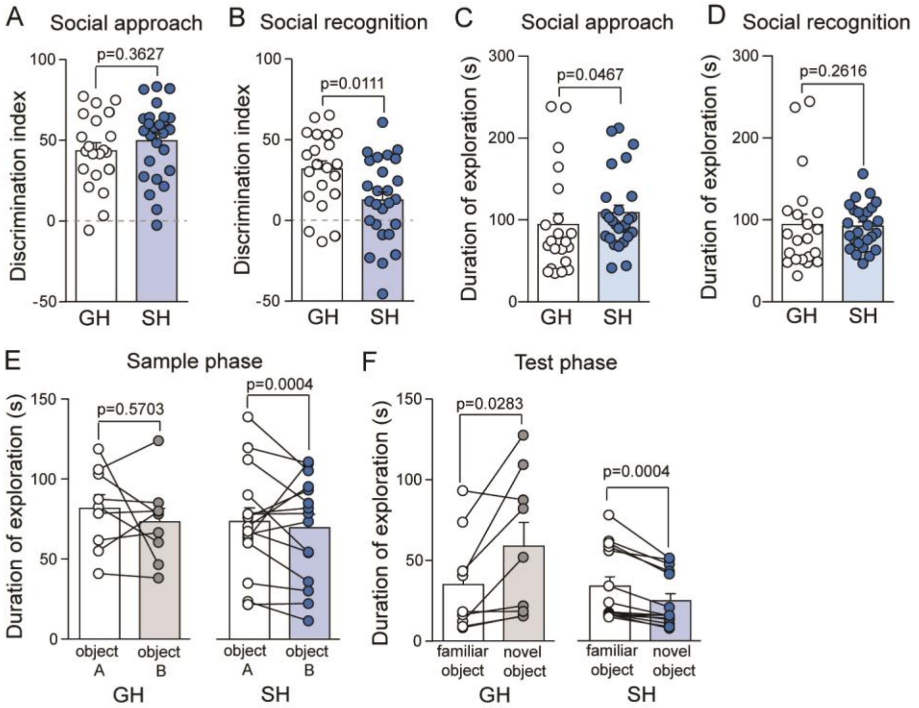

Supplemental Figure 1. Single housing has no effect on total exploration time during social approach or social recognition. Discrimination indexes for the social approach (A) and social recognition phases of the Three-Chamber Task of GH and SH mice. Total time of exploration in social approach (**C**) and for the social recognition phase (D) of GH and SH. (**E**) Time spent exploring objects A and B by GH (left) and SH (right) mice in the sample phase of the Novel Object Recognition Task. (**F**) Time spent exploring the familiar and novel objects by GH (left) and SH (right) mice in the test phase of the Novel Object Recognition Task.

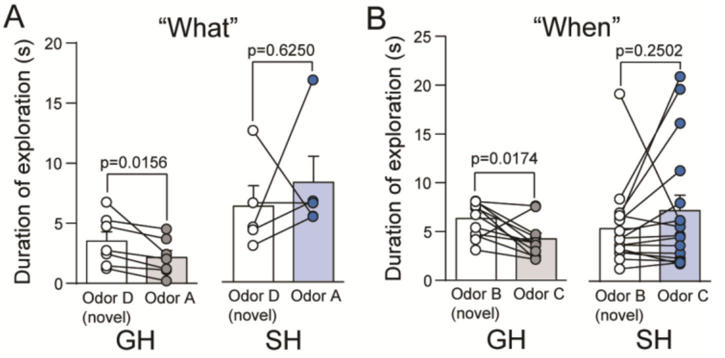

Supplemental Figure 2. Single housing reduces the proportional time spent exploring novel cues. (A) In the episodic "What" odor task, GH animals (*left*) spent significantly more time exploring the novel odor D vs. familiar odor A whereas SH animals (*right*) did not. (**B**) In the "When" odor task, GH animals (*left*) explored the less recent, more novel, odor B for a greater amount of time vs. odor C. By contrast there's no group difference in the time spent exploring odor B vs odor C for SH animals (*right*).

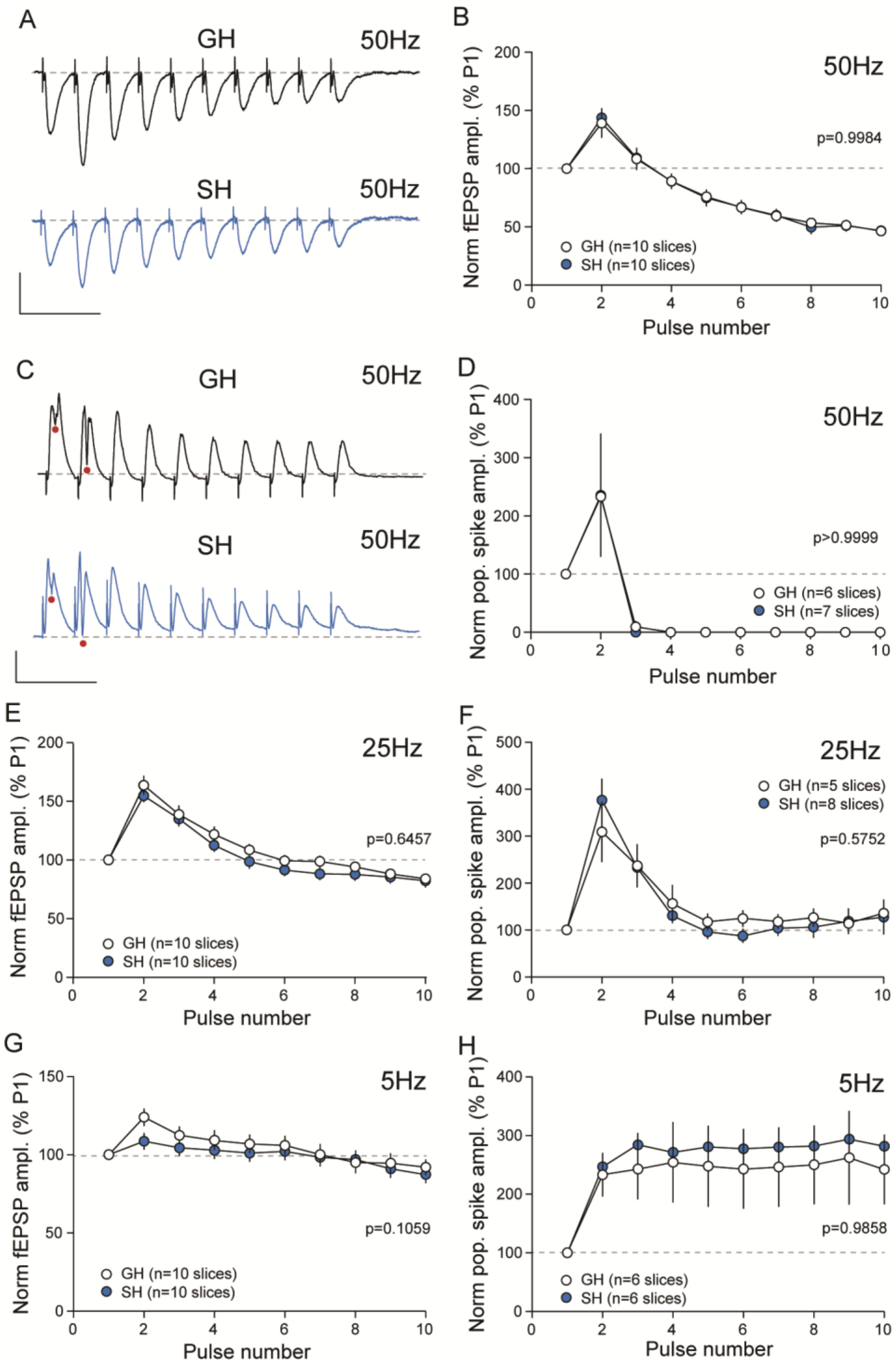

Supplemental Figure 3. Frequency-dependent operations of the LPP-DG synapse are unaffected by single housing. Representative traces recorded from the DG outer molecular layer (OML; **A**) and granule cell (GC; **C**) layer of GH (top) and SH (bottom) slices in response to

LPP stimulation with a brief (10 pulse) 50Hz train. Scale bars: **A**. y = 1mV, x = 50ms; **C**. y = 2mV, x = 50ms. Graphs summarizing the within train facilitation of the fEPSP amplitude (**B**) and the population spike amplitude (**D**) for the 50Hz stimulation for each group. Graphs summarizing the within-train facilitation of the fEPSP amplitude and population spike amplitude following LPP stimulation at 25Hz (**E**, **F**) and 5Hz (**G**, **H**) recorded from GH and SH slices.

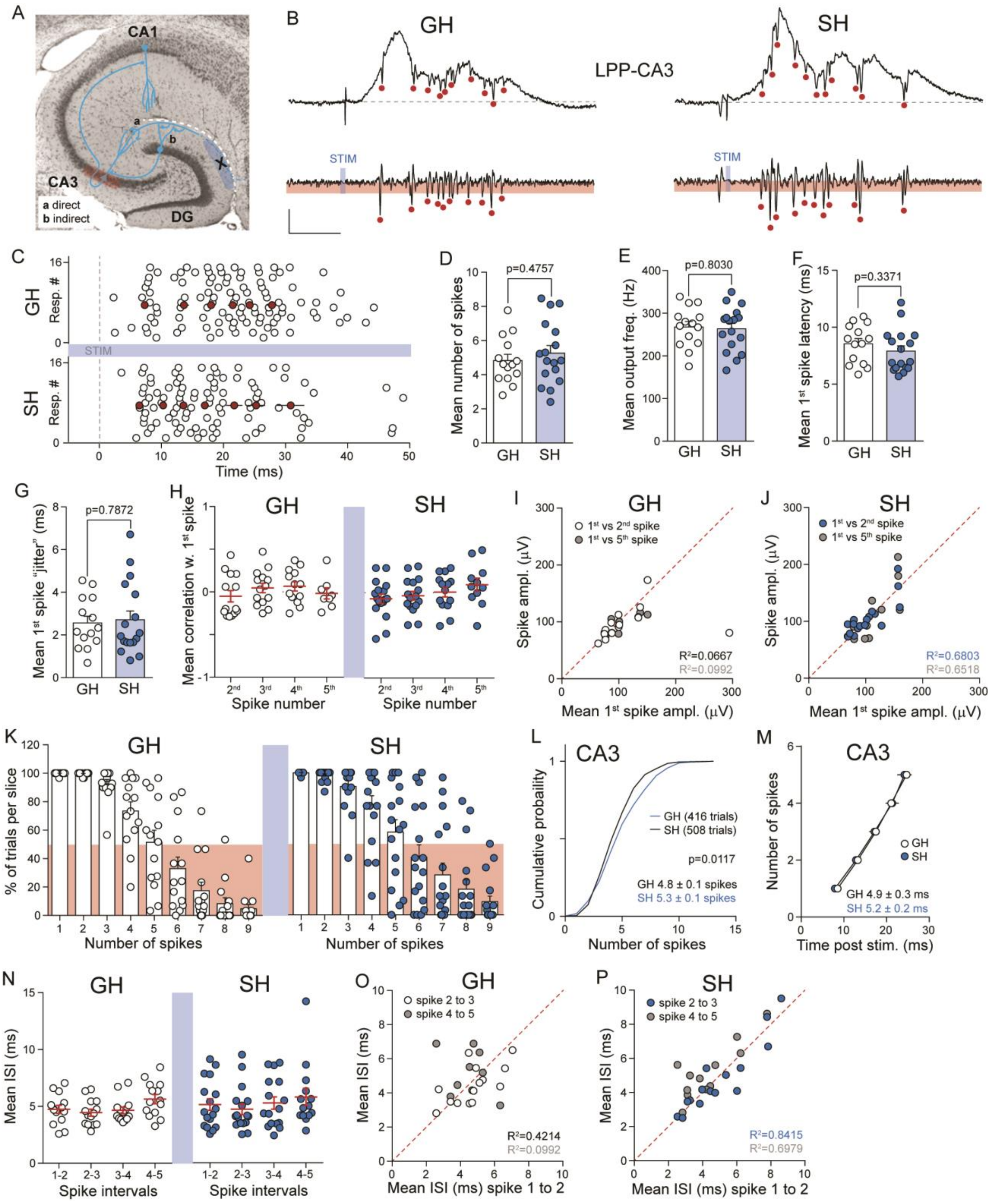

Supplemental Figure 4. Exposure to single housing has no effect upon CA3 spiking elicited by single-pulse LPP activation. (A) Nissl stain of a hippocampal slice showing the location of the stimulating electrode (x) in the LPP and the recording pipette in CA3 (red); the

direct and indirect sub-circuits are illustrated (a,b). (**B**) Exemplar raw (*top*) and filtered (*bottom*) CA3 responses to single-pulse LPP stimulation recorded from GH (*left*) and SH (*right*) slices; red dots identify single units in each trace (scale bars: y=0.2mV and 0.1mV, x=10ms). (**C**) Raster plots show the distribution of individual spike times within LPP-evoked CA3 response (15 consecutive pulses; open circles) in representative slices from GH (*top*) and SH (*bottom*) mice. The mean output for each slice is shown (red circles). Bar graphs summarizing the mean number (**D**), mean output frequency (**E**), mean 1st spike latency (**F**) and the associated jitter (**G**) of spikes associated with LPP evoked CA3 responses in GH and SH slices. (**H**) Graphs summarizing the mean correlation of the 1st spike amplitude with those of the subsequent 4 spikes in CA3 following single-pulse LPP activation in GH (left; open circles) and SH (right; blue circles) slices. Scatter plots with unity line (red dashed) summarizing the correlation of the 1st spike amplitude with those of the 2nd and 5th spikes recorded from CA3a in response to single pulse LPP activation in GH (**I**) and SH (**J**) slices. **(K)** Graphs show the proportion (%) of LPP-evoked CA3 responses containing 1 to 9 spikes for each GH (left) and SH (right) slice; bars show mean ± SEM for each number of spike. (**L**) Cumulative probability plot showing the difference in the distribution of spike numbers per response recorded from GH (416 trials) and SH (508 trials) slices. (**M**) Graph showing the mean number of spikes along with their temporal distribution within the LPP-evoked CA3 response in GH and SH slices. (**N**) Graphs showing the mean inter-spike interval (ISI) between successive spikes (spikes 1 to 5) across the LPP-evoked CA3 waveform for each GH (left) and SH (right) slice. Scatter plots (unity line: red dashed) showing the relationships between the mean ISI of the 1st and 2nd CA3 spikes with those of the 2nd and 3rd spikes and 4th and 5th spikes in response to single pulse LPP stimulation in GH (**O**) and SH (**P**) slices.

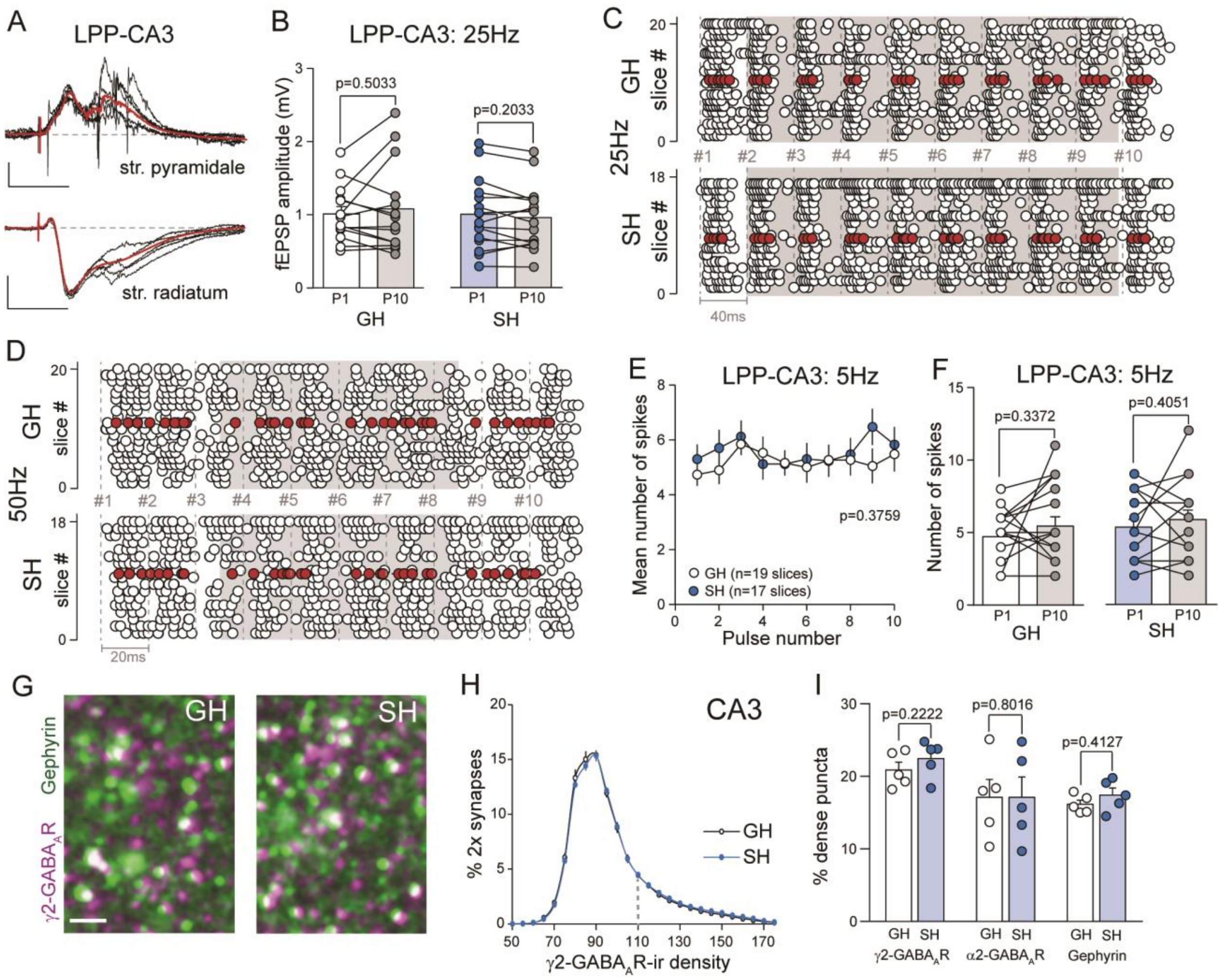

Supplemental Figure 5. Frequency-dependent operations in CA3 are selectively impaired in SH animals. (**A**) Representative LPP-evoked fEPSPs recorded from CA3 str. pyramidale and str. radiatum of a GH slice. The ensemble average fEPSP for each is shown in red (scale bars: y = 0.25mV, x = 20ms). (**B**) The amplitude of fEPSPs elicited by the 1st and 10th pulse of a 25Hz stimulation train in GH (*left*) and SH (*right*) hippocampal slices. (**C,D**) Raster plots showing the distribution of LPP-evoked CA3 spike times (open circles) elicited during brief (10 pulses) 25Hz (**C**) and 50Hz (**D**) stimulation trains in slices from GH (*top*) and SH (*bottom*) mice. In each, the mean (± SEM) spike output for all slices is shown (red circles). (**E**) Graph summarizing the mean number of LPP-evoked CA3 spikes in response to each pulse of a 5Hz (10 pulse) train in GH (open circles) and SH (blue circles) slices. (**F**) Number of CA3 spikes elicited on the 1st and

10th stimulation pulse of a 5Hz train in GH and SH slices. (**G**) Representative images showing immunofluorescence localization of gephyrin (green) with the γ2 subunit of the $GABA_AR$ (magenta) in CA3 str. radiatum of GH (left) and SH (right) slices (calibration bar, 2μm). (**H**) Histogram shows the density frequency distributions for γ2-ir co-localized with gephyrin ('2x synapses') in CA3 str. radiatum of GH and SH slices; profiles with labeling densities ≥ 110 (dotted line) were considered densely labeled. (**I**) Percentages (%) of double-labeled synapses (mean ± SEM) with dense immunolabeling for $GABA_AR$ γ2, $GABA_AR$ α2, and gephyrin proteins were not different between GH and SH groups.

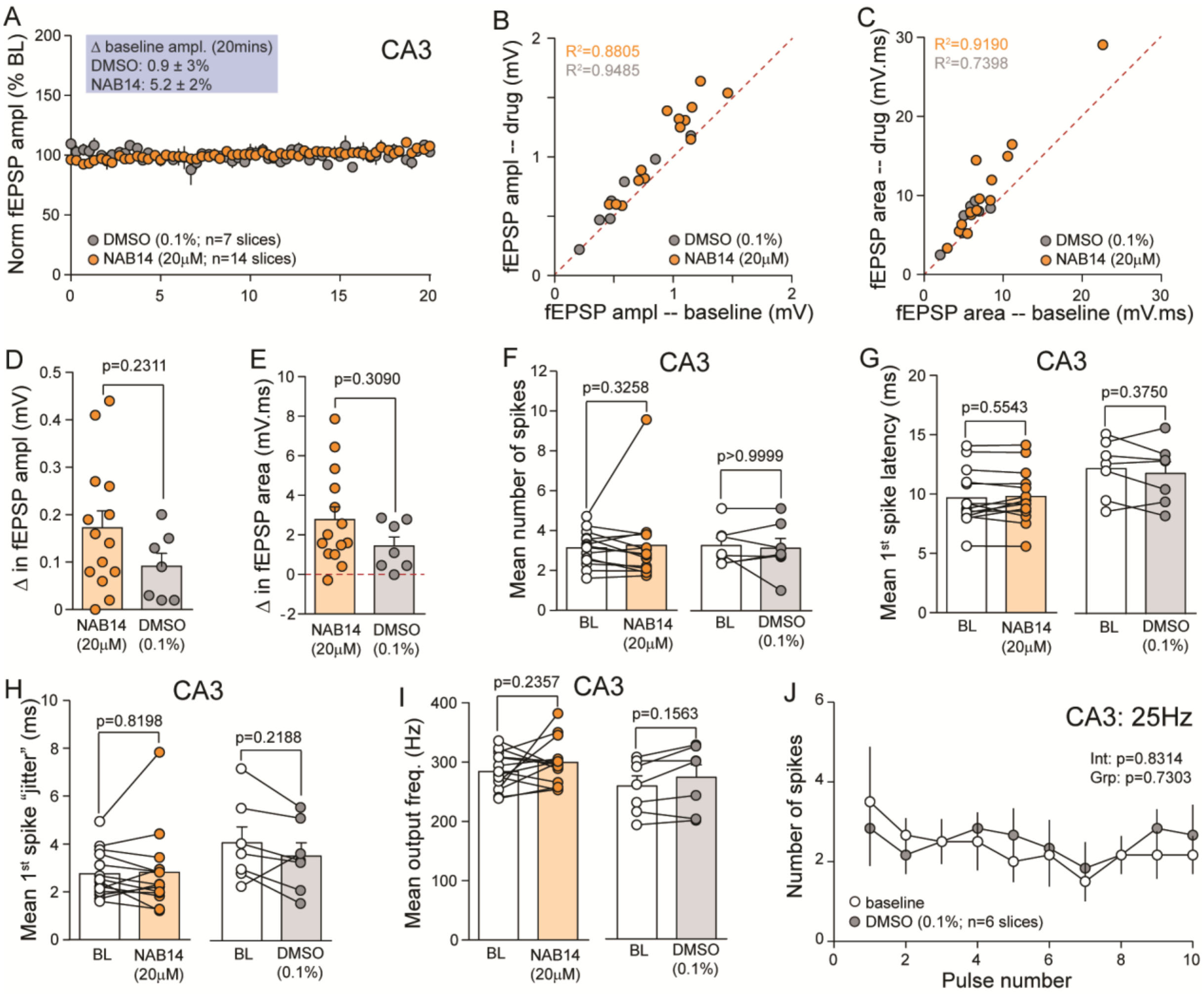

Supplemental Figure 6. NAB14 has no effect upon CA3 response to single pulse LPP activation. (A). Graph showing the mean normalized fEPSP amplitude elicited by single pulse LPP activation across the baseline period (20 minutes) for the NAB14 or DMSO treatment groups. The mean % change (Δ) for each is shown in the inset. Graph showing the fEPSP amplitude (**B**) and area (**C**) before and after treatment with NAB14 or DMSO. Graph showing the change in fEPSP amplitude (**D**) and area (**E**) following treatment with NAB14 or DMSO. Graph showing the effect of NAB14 or DMSO treatment upon the number of CA3 spikes (**F**), mean 1st spike latency (**G**), 1st spike "jitter" (**H**) and mean spike output frequency (**I**) elicited by single pulse LPP activation. (**J**) Graph summarizing the mean number of LPP-evoked CA3 spikes in response to each pulse of a 25Hz (10 pulse) train before and after DMSO treatment.

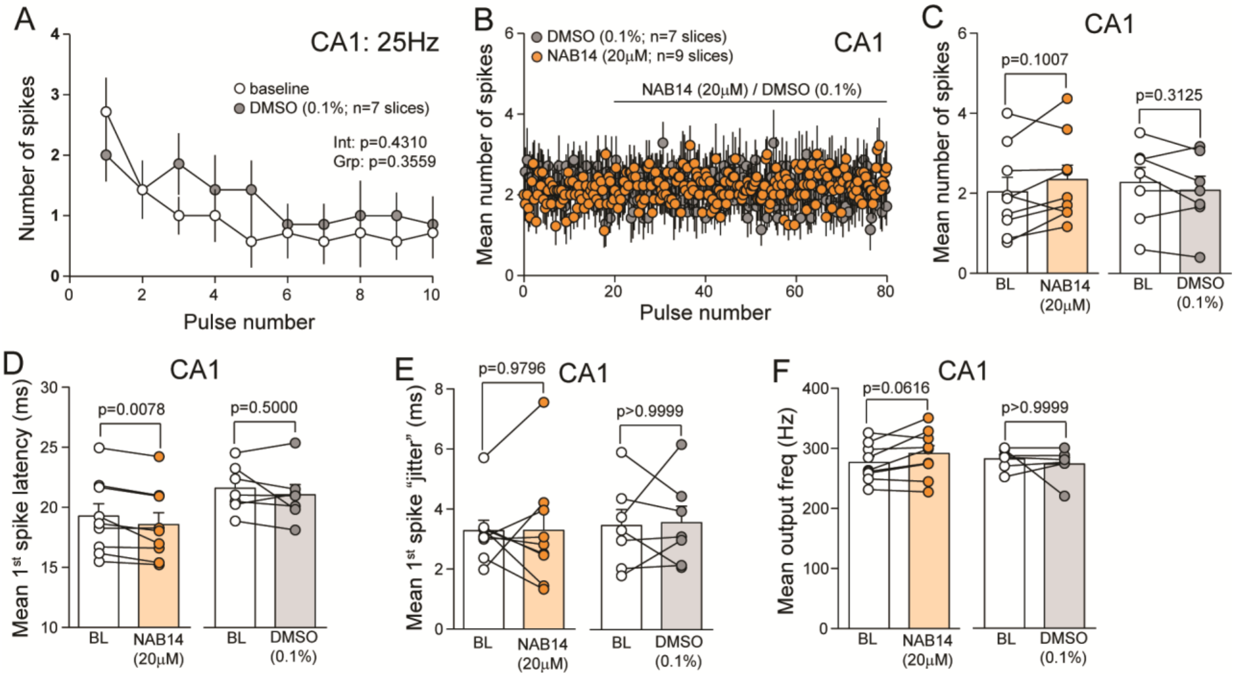

Supplemental Figure 7. NAB14 has minimal effects upon CA1 response to single pulse LPP activation. (A). Graph summarizing the mean number of LPP-evoked CA1 spikes in response to each pulse of a 25Hz (10 pulse) train before and after DMSO treatment. (**B**). Graph showing the mean number of LPP-evoked CA1 spikes prior to, and during 60-min bath application of NAB14 or DMSO. Graph showing the effect of NAB14 or DMSO treatment upon the number of CA1 spikes (**C**), mean 1st spike latency (**D**), 1st spike "jitter" (**E**) and mean spike output frequency (**F**) elicited by single pulse LPP activation.

Supplemental Tables

Supplemental Table 1. Detailed Statistics

A. Main Figures

Fig #	Experiment	Group	Mean ± SEM	n	Statistical test	Norm. test
**1B**	Social Approach	Empty vs<br>Mouse	GH: -0.57 ± 0.1<br>SH: -0.31 ± 0.1	GH: 21<br>SH: 26	GH: Obj. vs mouse: p<0.0001<br>SH: Obj. vs mouse: p<0.0001<br>Wilcoxon matched-pairs	GH: p=0.0011<br>SH: p=0.0008
**1C**	Social Recognition	Familiar vs<br>Novel	GH: -0.42 ± 0.04<br>SH: -0.05 ± 0.04	GH: 21<br>SH: 26	GH: fam. vs novel: p=0.0002<br>Paired Student's t test<br>SH: fam. vs novel: p=0.0435<br>Wilcoxon matched-pairs	GH: p=0.1511<br>SH: p=0.0905
**1D**	Novel Object<br>Recognition Test	Familiar vs<br>Novel	GH: 62.99 ± 3.4<br>SH: 42.24 ± 1.2	GH: 9<br>SH: 15	GH vs. SH: p<0.0001<br>Unpaired Student t test	GH: p=0.9411<br>SH: p=0.9240
**1**	Tail Suspension Test	Static vs<br>movement	GH: 148.20 ± 0.8<br>SH: 212.93 ± 1.2	GH: 12<br>SH: 20	GH vs. SH: p<0.0001<br>Unpaired Student t test	GH: p=0.9171<br>SH: p=0.4798
**1F**	Forced Swim Test	Static vs<br>movement	GH: 155.30±7.8<br>SH: 181.0±5.0	GH: 13<br>SH: 15	GH vs. SH: p=0.0044<br>Mann-Whitney test	GH: p=0.5453<br>SH: p=0.0471
**1G**	Palatable Food<br>Consumption		GH: 0.20±0.02<br>SH: 0.32±0.02	GH: 12<br>SH: 11	GH vs. SH: p=0.0021<br>Unpaired Student t test	GH: p=0.7961<br>SH: p=0.4135
**1I Left**	Episodic "What"<br>odor task	Familar vs<br>Novel	GH: 28.93±9.0<br>SH: -13.51±16.1	GH: 7<br>SH: 5	GH vs. SH: p=0.0480<br>Mann-Whitney test	GH: n/a<br>SH: n/a
**1I**<br>**Right**	Episodic "When"<br>odor task	Recent vs<br>older<br>*(odor B vs<br>odorC)	GH: 21.33±6.7<br>SH:-7.81±6.8	GH: 11<br>SH: 16	GH vs. SH: p=0.0071<br>Unpaired Student t test	GH: p=0.1366<br>SH: p=0.7207
**1J**	Episodic Memory<br>task	Familar vs<br>Novel	GH: 32.76± 7.6<br>SH:-14.10±5.8	GH: 4<br>SH: 13	GH vs. SH: p=0.0034<br>Mann-Whitney test	GH: n/a<br>SH: p=0.6472
**2B**	LHb fos expression	Fos cell<br>count<br>LHb	GH: 2.6±0.4<br>SH: 10.0±1.7	GH: 5<br>SH: 5	GH vs. SH: p=0.0079<br>Mann-Whitney test	GH: n/a<br>SH: n/a
**2B**	MHb fos expression	Fos cell<br>count	GH: 0.4±0.1<br>SH: 0.6±0.2	GH: 5<br>SH: 5	GH vs. SH: p=0.4879<br>Mann-Whitney test	GH: n/a<br>SH: n/a

		MHb				
**2E Left**	LHb (lateral division) fos expression	Fos cell count		GH: 5<br>SH: 5	GH vs. SH:<br>Interaction: $F_{(7, 56)}$=0.5693; p<0.0001<br>Group: $F_{(1,8)}$= 11.43; p=0.0096<br>Section: $F_{(7, 56)}$=7.544; p<0.0001<br>Two way RM ANOVA	P=0.9995
**2E Right**	LHb (medial division) fos expression	Fos cell count		GH: 5<br>SH: 5	GH vs. SH:<br>Interaction: $F_{(7, 56)}$=2.708; p=0.0172<br>Group: $F_{(1, 8)}$=17.24; p=0.0032<br>Section: $F_{(7, 56)}$=4.019; p=0.0013<br>Two way RM ANOVA	P=09982
**3C Left**	Mean CV slope dendritic fEPSP (Str, radiatum)	1St vs 2nd	GH 1st: 16.4 ± 2.3<br>GH 2nd: 48.3 ± 5.8<br>SH 1st: 15.7 ± 2.5<br>SH 2nd: 44.6 ± 7.6	GH: 14 slices<br>SH: 14 slices	GH 1St vs 2nd: p=0.0002<br>SH 1St vs 2nd: p=0.0017<br>Paired Student's t test	GH: p=0.4122<br>SH: p=0.0837
**3C Right**	Mean CV amplitude somatic fEPSP (Str, pyramidale)	1St vs 2nd	GH 1st: 20.3 ± 2.9<br>GH 2nd: 46.8 ± 4.5<br>SH 1st: 19.2 ± 3.5<br>SH 2nd: 43.8 ± 3.4	GH: 16 slices<br>SH: 14 slices	GH 1St vs 2nd p=0.0003<br>SH 1St vs 2nd p=0.0005<br>Paired Student's t test	GH: p=0.3604<br>SH: p=0.6279
**3E Left**	% CA1 responses in initial EPSP	GH vs SH	GH: 4.2 ± 1.3<br>SH: 4.0 ± 1.0	GH: 25 slices<br>SH: 25 slices	p=0.8336<br>Mann-Whitney test	GH: p<0.0001<br>SH: p=0.0469
**3E Right**	% CA1 responses in second EPSP	GH vs SH	GH: 93.7 ± 1.4<br>SH: 90.5 ± 3.0	GH: 25 slices<br>SH: 25 slices	p=0.8917<br>Mann-Whitney test	GH: p=0.2062<br>SH: p<0.0001
**3F**	Mean number of CA1 spikes	GH vs SH	GH: 2.6±0.2<br>SH: 3.1±0.3	GH: 25 slices<br>SH: 25 slices	GH vs SH: p=0.2986<br>Unpaired Student's t test	GH: p=0.3445<br>SH: p=0.3408
**3G**	Latency to first spike CA1	GH vs SH	GH: 20.0±0.9<br>SH: 19.6±0.6	GH: 25 slices<br>SH: 25 slices	GH vs SH: p=0.7239<br>Unpaired Student's t test	GH: p=0.8375<br>SH: p=0.4934
**3H**	Mean first spike	GH vs SH	GH: 4.2±0.4	GH: 25	GH vs SH: p=0.5609	GH: p=0.3783

	'jitter" CA1		SH: 3.9±0.3	slices<br>SH: 25<br>slices	Mann-Whitney test	SH: p=0.0007
**3J**	Distribution of spike per response CA1	GH vs SH	GH: 2.6±0.2<br>SH: 3.1±0.1	GH: 738 trials<br>SH: 710 trials	GH vs SH: p<0.0001<br>Kolmogorov–Smirnov Test	GH: p<0.0001<br>SH: p<0.0001
**3L**	ISI of CA1 spikes	GH vs SH	GH S1-2: 4.4±0.3<br>GH S2-3: 4.2±0.2<br>SH S1-2: 4.4±0.3<br>SH S2-3: 3.9±0.3		GH vs SH:<br>Interaction: $F_{(1, 25)}$=0.5193; p=0.4778<br>Group: $F_{(1, 25)}$=0.2614; p=0.6136<br>Time: $F_{(1, 25)}$=2.454; p=0.1298<br>Two way RM ANOVA	P = 0.9973
**3M**	Mean output frequency of CA1 spikes	GH vs SH	GH: 260.6±7.3<br>SH: 275.7±10.1	GH: 25 slices<br>SH: 25 slices	GH vs SH: p=0.2324<br>Unpaired Student's t test	GH: p=0.0930<br>SH: p=0.3829
**3N**	Mean CV 1st spike amplitude (CA1)	GH vs SH	GH: 37.7±2.8<br>SH: 39.3±2.3	GH: 25 slices<br>SH: 25 slices	GH vs SH: p=0.4496<br>Mann-Whitney test	GH: p=0.0095<br>SH: p=0.0955
**4C**	Mean spike # CA1 (5Hz train)	GH vs SH		GH: 23 slices<br>SH: 24 slices	GH vs SH:<br>Interaction: $F_{(9, 405)}$=1.109, p=0.3550<br>Group: $F_{(1, 45)}$=1.109, p=0.4027<br>Time: $F_{(9, 405)}$=3.034, p=0.0016<br>Two way RM ANOVA	P = 0.9974
**4D**	Mean spike # CA1 P1 vs P10 GH & SH (5Hz train)	P1 vs P10	GH:<br>P1=3.1 ± 0.4<br>P10=3.2 ± 0.5<br>SH:<br>P1=3.2 ± 0.4<br>P10=4.6 ± 0.8	GH: 23 slices<br>SH: 24 slices	GH P1 vs P10 p=0.9248<br>Paired Student's t test<br>SH P1 vs P10 p=0.2764<br>Wilcoxon matched-pairs	GH: p=0.4736<br>SH: p=0.0005
**4G**	Mean spike # CA1 (theta-gamma train)	GH vs SH		GH: 23 slices<br>SH: 20 slices	GH vs SH:<br>Interaction: $F_{(4, 164)}$=3.278, p=0.0129<br>Group: $F_{(1, 41)}$=2.184, p=0.1471<br>Time: $F_{(4, 164)}$=7.613, p<0.0001<br>Two way RM ANOVA	P = 0.9974
**4H**	Mean Δ spike #CA1 (theta-gamma train)	GH vs SH		GH: 23 slices<br>SH: 20	GH vs SH:<br>Interaction: $F_{(4, 164)}$=3.278, p=0.0129<br>Group: $F_{(1, 41)}$=7.665, p=0.0084<br>Time: $F_{(4, 164)}$=7.613, p<0.0001<br>Two way RM ANOVA	P = 0.9974

				slices		
**4K**	Mean spike # CA1<br>(50Hz train)	GH vs SH		GH: 28<br>slices<br>SH: 21<br>slices	GH vs SH:<br>Interaction: $F_{(3, 141)}$=2.823, p=0.0411<br>Group: $F_{(1, 47)}$=8.125, p=0.0065<br>Time: $F_{(3, 141)}$=10.38, p<0.0001<br>Two way RM ANOVA	P = 0.9960
**4L**	Mean spike # CA1<br>Epoch 1 vs Epoch 4<br>GH & SH<br>(50Hz train)	E1 vs E4	GH:<br>E1=3.4 ± 0.3<br>E4=1.5 ± 0.3<br>SH:<br>E1=3.6 ± 0.4<br>E4=3.1 ± 0.5	GH: 28<br>slices<br>SH: 21<br>slices	GH E1 vs E4 p<0.0001<br>SH E1 vs E4 p=0.3003<br>Paired Student's t test	GH: p=0.9133<br>SH: p=0.9300
**4O**	Mean spike # CA1<br>(25Hz train)	GH vs SH		GH: 24<br>slices<br>SH: 23<br>slices	GH vs SH:<br>Interaction: $F_{(9, 405)}$=2.345, p=0.0138<br>Group: $F_{(1, 45)}$=3.715, p=0.0603<br>Time: $F_{(9, 405)}$=7.151, p<0.0001<br>Two way RM ANOVA	P = 0.9974
**4P**	Mean spike # CA1<br>P1 vs P10 GH & SH<br>(25Hz train)	P1 vs P10	GH:<br>P1=3.1 ± 0.3<br>P10=0.9 ± 0.2<br>SH:<br>P1=2.5 ± 0.4<br>P10=2.4 ± 0.5	GH: 24<br>slices<br>SH: 23<br>slices	GH P1 vs P10 p<0.0001<br>Paired Student's t test<br>SH P1 vs P10 p=0.4346<br>Wilcoxon matched-pairs	GH: p=0.7493<br>SH: p=0.0003
**5B**	Facilitation of norm.<br>CA3 fEPSP amplitude<br>(25Hz train)	GH vs SH		GH: 14<br>slices<br>SH: 16<br>slices	GH vs SH:<br>Interaction: $F_{(9, 252)}$=1.549, p=0.1313<br>Group: $F_{(1, 28)}$=0.4484, p=0.5086<br>Time: $F_{(9, 252)}$=15.03, p<0.0001<br>Two way RM ANOVA	P = 0.9973
**5E**	Mean spike # CA3<br>(25Hz train)	GH vs SH		GH: 20<br>slices<br>SH: 17<br>slices	GH vs SH:<br>Interaction: $F_{(9, 315)}$=2.139, p=0.0261<br>Group: $F_{(1, 35)}$=1.331, p=0.2564<br>Time: $F_{(9, 315)}$=1.711, p=0.0792<br>Two way RM ANOVA	P = 0.9961
**5F**	Mean spike # CA3<br>P1 vs P10 GH & SH<br>(25Hz train)	P1 vs P10	GH:<br>P1=5.95 ± 0.6<br>P10=3.65 ± 0.3<br>SH:<br>P1=5.06 ± 0.6<br>P10=5.24 ± 0.7	GH: 20<br>slices<br>SH: 17<br>slices	GH P1 vs P10 p=0.0051<br>SH P1 vs P10 p=0.7660<br>Paired Student's t test	GH: p=0.1453<br>SH: p=0.7559
**5J**	Mean spike # CA3<br>(50Hz train)	GH vs SH		GH: 20<br>slices<br>SH: 17<br>slices	GH vs SH:<br>Interaction: $F_{(3, 105)}$=0.9994, p=0.3963<br>Group: $F_{(1, 35)}$=0.2205, p=0.3563<br>Time: $F_{(3, 105)}$=1.091, p=0.3963<br>Two way RM ANOVA	P = 0.9975

**5K**	Mean spike # CA3 (theta-gamma train)	GH vs SH		GH: 18 slices SH: 16 slices	GH vs SH: Interaction: $F_{(4, 128)}$=1.988, p=0.1002 Group: $F_{(1, 32)}$=0.8909, p=0.3523 Time: $F_{(4, 128)}$=0.8845, p=0.4753 Two way RM ANOVA	P = 0.9975
**6B**	Mean CA3 fEPSP decay τ	BL vs NAB14	BL: 3.97 ± 0.3 NAB: 4.99 ± 0.5	14 slices	BL vs NAB14: p=0.0001 Wilcoxon matched-pairs	p=0.0181
**6C**	CA3 decay τ	BL vs NAB14 or DMSO		NAB: 14 slices DMSO: 7 slices	NAB14 : R^2=0.6472 DMSO: R^2=0.8402 Linear Regression	
**6D**	Mean Δ in CA3 fEPSP decay τ	NAB14 vs DMSO	NAB: 1.02 ± 0.3 DMSO: -0.04 ± 0.7	NAB: 14 slices DMSO: 7 slices	NAB14 vs DMSO: p=0.0031 Mann-Whitney test	NAB: p=0.0181 DMSO: n/a
**6G**	Facilitation of norm. CA3 fEPSP amplitude (25Hz train)	BL vs NAB14		NAB: 12 slices	BL vs NAB14: Interaction: $F_{(9, 99)}$=1.051, p=0.4057 Group: $F_{(1, 11)}$=0.0060, p=0.9396 Time: $F_{(9, 99)}$=7.149, p=0<0.0001 Two way RM ANOVA	P = 0.9981
**6I**	Mean spike # CA3 (25Hz train)	BL vs NAB14		12 slices	BL vs NAB14: Interaction: $F_{(9, 99)}$=1.996, p=0.0475 Group: $F_{(1, 11)}$=12.16, p=0.0051 Time: $F_{(9, 99)}$=2.270, p=0.0234 Two way RM ANOVA	P = 0.9853
**6K**	Mean spike # CA1 (25Hz train)	BL vs NAB14		9 slices	BL vs NAB14: Interaction: $F_{(9, 72)}$=1.532, p=0.1533 Group: $F_{(1, 8)}$=28.59, p=0.0007 Time: $F_{(9, 72)}$=3.364, p=0.0013 Two way RM ANOVA	P = 0.9767

B. Supplemental Figures

**S1A**	Social Approach 3 Chamber Test	Familiar vs Novel	GH: 43.47 ± 5.0 SH: 49.64 ± 4.5	GH: 21 SH: 26	GH vs SH: p=0.3627 Unpaired Student's t test	GH: p=0.8070 SH: p=0.3502
**S1B**	Social Recognition 3 Chamber Test	Familiar vs Novel	GH: 31.72 ± 5.1 SH: 12.59 ± 5.0	GH: 21 SH: 26	GH vs SH: p=0.0111 Unpaired Student's t test	GH: p=0.4804 SH: p=0.7577
**S1C**	Social Approach	Empty vs	GH: 94.11±13.8	GH: 21	GH vs. SH: p=0.0467	GH: p=0.0247

		Mouse	SH:108.6±9.1	SH: 26	Mann-Whitney test	SH: p=0.0818
**S1D**	Social Recognition	Familiar vs Novel	GH: 94.00±12.8<br>SH:92.14±5.3	GH: 21<br>SH: 26	GH vs. SH: p=0.2616<br>Mann-Whitney test	GH: p=0.0025<br>SH: p=0.6767
**S1E**	NOR - Sample	Object A vs Object B	Sample GH<br>Obj A: 81.58 ± 8.5<br>Obj B: 72.80 ± 8.3<br>Sample SH<br>Obj A: 72.80 ± 8.5<br>Obj B: 68.91 ± 8.4	GH: 9<br>SH: 15	GH: p=0.5703<br>Wilcoxon matched pairs<br>SH: 0.5139<br>Paired Student's t test	GH: p=0.0411<br>SH: p=0.4084
**S1F**	NOR - Test	Familiar vs Novel	Test GH<br>Fam: 35.00 ± 10.2<br>Nov: 58.86 ± 14.6<br>Test SH<br>Obj A: 33.64 ± 5.8<br>Obj B: 24.75 ± 4.3	GH: 9<br>SH: 15	GH: p=0.0283<br>SH: p=0.0004<br>Paired Student's t test	GH: p=0.4390<br>SH: p=0.1477
**S2A**	Episodic "What" odor task – GH & SH	Familiar vs Novel	GH<br>Fam: 2.17 ± 0.6<br>Nov: 3.52 ± 0.8<br>SH<br>Fam: 8.09 ± 1.7<br>Nov: 6.14 ± 1.7	GH: 7<br>SH: 5	GH: familiar vs novel p=0.0156<br>SH: familiar vs novel p=0.6250<br>Wilcoxon matched pairs	GH: n/a<br>SH: n/a
**S2B**	Episodic "When" odor task – GH & SH	Familiar vs Novel	GH<br>Fam: 4.12 ± 0.6<br>Nov: 6.19 ± 0.6<br>SH<br>Fam: 7.3 ± 1.6<br>Nov: 5.45 ± 1.0	GH: 11<br>SH: 16	GH: familiar vs novel p=0.0174<br>SH: familiar vs novel p=0.2502<br>Paired Student's t test	GH: p=0.1581<br>SH: p=0.2658
**S3B**	LPP-DG: facilitation fEPSP ampl. (norm) (50Hz train)	GH vs. SH		GH: 10 slices<br>SH: 10 slices	GH vs SH:<br>Interaction: $F_{(9,162)}$=0.1405, p=0.9984<br>Group: $F_{(1,18)}$=0.0005, p=0.9828<br>Time: $F_{(9,162)}$=125.7, p<0.0001<br>Two way RM ANOVA	P = 0.9981
**S3D**	LPP-DG: facilitation pop. spike ampl. (norm) (50Hz train)	GH vs. SH		GH: 6 slices<br>SH: 7 slices	GH vs SH:<br>Interaction: $F_{(9, 99)}$=0.0066, p>0.9999<br>Group: $F_{(1,11)}$=0.0033, p=0.9551<br>Time: $F_{(9,99)}$=16.03, p<0.0001<br>Two way RM ANOVA	P = 0.9975
**S3E**	LPP-DG: facilitation fEPSP ampl. (norm) (25Hz train)	GH vs. SH		GH: 10 slices<br>SH: 10 slices	GH vs SH:<br>Interaction: $F_{(9,162)}$=0.7685, p=0.6457<br>Group: $F_{(1,18)}$=0.9038, p=0.3543<br>Time: $F_{(9,162)}$=129.3, p<0.0001<br>Two way RM ANOVA	P = 0.9981
**S3F**	LPP-DG: facilitation pop. spike ampl. (norm)	GH vs. SH		GH: 5 slices<br>SH: 8	GH vs SH:<br>Interaction: $F_{(9,99)}$=0.8467, p=0.5752<br>Group: $F_{(1,11)}$=0.0275, p=0.8713<br>Time: $F_{(9,99)}$=24.6, p<0.0001	P = 0.9974

	(25Hz train)			slices	Two way RM ANOVA	
**S3G**	LPP-DG: facilitation fEPSP ampl. (norm) (5Hz train)	GH vs. SH		GH: 10 slices SH: 10 slices	GH vs SH: Interaction: $F_{(9,162)}$=1.648, p=0.1059 Group: $F_{(1,18)}$=0.4615, p=0.5056 Time: $F_{(9,162)}$=17.6, p<0.0001 Two way ANOVA	P = 0.9981
**S3H**	LPP-DG: facilitation pop. spike ampl. (norm) (5Hz train)	GH vs. SH		GH: 6 slices SH: 6 slices	GH vs SH: $F_{(9, 90)}$=0.2492, p=0.9858 Interaction: $F_{(9, 90)}$=0.2492, p=0.9858 Group: $F_{(1,10)}$=0.2301, p=0.6418 Time: $F_{(9,162)}$=15.5, p<0.0001 Two way RM ANOVA	P = 0.9980
**S4D**	Mean spike # CA3	GH vs SH	GH: 4.8 ± 0.4 SH: 5.3 ± 0.4	GH: 14 slices SH: 17 slices	GH vs. SH: p=0.4757 Unpaired Student's t test	GH: p=0.4368 SH: p=0.5745
**S4E**	Mean CA3 output frequency	GH vs SH	GH: 267.9 ± 12.5 SH: 263.4 ± 12.6	GH: 14 slices SH: 17 slices	GH vs. SH: p=0.8030 Unpaired Student t test	GH: p=0.8296 SH: p=0.7104
**S4F**	Latency to 1st spike CA3	GH vs SH	GH: 8.5±0.5 SH: 7.9±0.5	GH: 14 slices SH: 17 slices	GH vs. SH: p=0.3371 Unpaired Student t test	GH: p=0.2630 SH: p=0.2279
**S4G**	Mean first spike 'jitter" CA3	GH vs SH	GH: 2.6±0.3 SH: 2.7±0.4	GH: 14 slices SH: 17 slices	GH vs. SH: p=0.7872 Unpaired Student t test	GH: p=0.5923 SH: p=0.1242
**S4I**	CA3 spike ampl - GH	1st spike vs 2nd 1st spike vs 5th		GH: 14 slices	1st spike vs 2nd : R^2=0.0667 1st spike vs 5th : R^2=0.0992 Linear Regression	
**S4J**	CA3 spike ampl - SH	1st spike vs 2nd 1st spike vs 5th		SH: 17 slices	1st spike vs 2nd : R^2=0.6803 1st spike vs 5th : R^2=0.6518 Linear Regression	
**S4K**	Proportion of trials	GH vs SH		GH: 14	GH vs SH:	P = 0.9973

	(%) containing 1-9 spikes in CA3			slices<br>SH: 17 slices	Interaction: $F_{(8, 232)}$=0.7135, p=0.6795<br>Group: $F_{(1, 29)}$=0.4881, p=0.4903<br>Time: $F_{(8, 232)}$=176, p<0.0001<br>Two way RM ANOVA	
**S4L**	Distribution of spike per response CA3	GH vs SH	GH: 4.8±0.1<br>SH: 5.3±0.1	GH: 416 trials<br>SH: 508 trials	GH vs SH: p=0.0117<br>Kolmogorov–Smirnov Test	GH: p<0.0001<br>SH: p<0.0001
**S4O**	ISI of CA3a Amplitude of fEPSPs -GH	1st-2nd vs 2nd-3rd<br>1st-2nd vs 4th-5th		GH: 14 slices	1st-2nd vs 2nd-3rd: R^2=0.4214<br>1st-2nd vs 4th-5th: R^2=0.0992<br>Linear Regression	
**S4P**	ISI of CA3a Amplitude of fEPSPs -SH	1st-2nd vs 2nd-3rd<br>1st-2nd vs 4th-5th		SH: 17 slices	1st-2nd vs 2nd-3rd: R^2=0.8415<br>1st-2nd vs 4th-5th: R^2=0.6975<br>Linear Regression	
**S5B**	Mean CA3 fEPSP ampl. P1 vs P10 GH & SH (25Hz train)	P1 vs P10	GH:<br>P1=1.01 ± 0.1<br>P10=1.08 ± 0.2<br>SH:<br>P1=1.00 ± 0.1<br>P10=0.96 ± 0.11	GH: 14 slices<br>SH: 16 slices	GH P1 vs P10 p=0.5033<br>SH P1 vs P10 p=0.2033<br>Paired Student's t test	GH: p=0.1231<br>SH: p=0.8502
**S5E**	Mean CA3 spike # (5Hz train)	GH vs SH		GH: 19 slices<br>SH: 17 slices	GH vs SH:<br>Interaction: $F_{(9, 306)}$=1.082, p=0.3759<br>Group: $F_{(1, 34)}$=0.2899, p=0.1143<br>Time: $F_{(9, 306)}$=1.600, p<0.0001<br>Two way RM ANOVA	P = 0.9969
**S5F**	Mean spike # CA3 P1 vs P10 GH & SH (5Hz train)	P1 vs P10	GH:<br>P1=4.74 ± 0.4<br>P10=5.47 ± 0.6<br>SH:<br>P1=5.29 ± 0.5<br>P10=5.82 ± 0.65	GH: 19 slices<br>SH: 17 slices	GH P1 vs P10 p=0.3372<br>Wilcoxon-matched pairs<br>SH P1 vs P10 p=0.4051<br>Paired Student's t test	GH: p=0.0017<br>SH: p=0.1390
**S5I Left**	% double-labeled synapses for $GABA_AR\gamma_2$	GH vs SH	GH: 20.9±1.1<br>SH: 22.46±1.1	GH: 5<br>SH: 5	GH vs SH: p=0.2222<br>Mann-Whitney test	GH: n/a<br>SH: n/a
**S5I Middle**	% double-labeled synapses for $GABA_AR$ a_2	GH vs SH	GH: 17.1±2.5<br>SH: 17.1±2.8	GH: 5<br>SH: 5	GH vs SH: p=0.8016<br>Mann-Whitney test	GH: n/a<br>SH: n/a

**S5I Right**	% double-labeled synapses for gephyrin	GH vs SH	GH: 16.2±0.6<br>SH: 17.4±1.0	GH: 5<br>SH: 5	GH vs SH: p=0.4127<br>Mann-Whitney test	GH: n/a<br>SH: n/a
**S6B**	CA3 fEPSP ampl	BL vs NAB14 or DMSO		NAB: 14 slices<br>DMSO: 7 slices	NAB14 : R^2=0.8805<br>DMSO: R^2=0.9485<br>Linear Regression	
**S6C**	CA3 fEPSP area	BL vs NAB14 or DMSO		NAB: 14 slices<br>DMSO: 7 slices	NAB14 : R^2=0.9190<br>DMSO: R^2=0.7398<br>Linear Regression	
**S6D**	Mean Δ in CA3 fEPSP ampl	NAB vs DMSO	NAB: 0.17± 0.04<br>DMSO: 0.09± 0.03	NAB: 14 slices<br>DMSO: 7 slices	NAB14 vs DMSO: p=0.2311<br>Mann-Whitney test	GH: p=0.3663<br>SH: n/a
**S6E**	Mean Δ in CA3 fEPSP area	NAB vs DMSO	NAB: 2.78 ± 0.6<br>DMSO: 1.43 ± 0.5	NAB: 14 slices<br>DMSO: 7 slices	NAB14 vs DMSO: p=0.3090<br>Mann-Whitney test	GH: p=0.2846<br>SH: n/a
**S6F**	Mean # CA3 spikes	BL vs NAB14 or DMSO	BL: 3.12 ± 0.2<br>NAB: 3.24 ± 0.5<br>BL: 3.31 ± 0.4<br>DMSO: 3.17 ± 0.5	NAB: 14 slices<br>DMSO: 7 slices	NAB14: p=0.3258<br>DMSO: p>0.9999<br>Wilcoxon matched-pairs	GH: p<0.0001<br>SH: n/a
**S6G**	Mean 1st spike latency CA3	BL vs NAB14 or DMSO	BL: 9.68 ± 0.6<br>NAB: 9.79 ± 0.6<br>BL: 12.24 ± 0.9<br>DMSO: 11.85 ±1.0	NAB: 14 slices<br>DMSO: 7 slices	NAB14: p=0.5543<br>Paired Student's t test<br>DMSO: p=0.3750<br>Wilcoxon matched-pairs	GH: p=0.8795<br>SH: n/a
**S6H**	Mean 1st spike "jitter" CA3	BL vs NAB14 or DMSO	BL: 2.75 ± 0.3<br>NAB: 2.81 ± 0.5<br>BL: 4.11 ± 0.7<br>DMSO: 3.55 ± 0.6	NAB: 14 slices<br>DMSO: 7 slices	NAB14: p=0.8198<br>DMSO: p=0.2188<br>Wilcoxon matched-pairs	GH: p=0.0004<br>SH: n/a
**S6I**	Mean CA3 output frequency	BL vs NAB14 or DMSO	BL: 284 ± 8<br>NAB: 300 ± 10<br>BL: 257 ± 17<br>DMSO: 271 ± 21	NAB: 14 slices<br>DMSO: 7 slices	NAB14: p=0.2357<br>Paired Student's t test<br>DMSO: p=0.1563<br>Wilcoxon matched-pairs	GH: p=0.0550<br>SH: n/a

**S6J**	Mean spike # CA3<br>(25Hz train)	BL vs<br>DMSO		6 slices	BL vs DMSO:<br>Interaction: $F_{(9, 45)}$=0.9307, p=0.8314<br>Group: $F_{(1, 5)}$=0.1330, p=0.7303<br>Time: $F_{(9, 45)}$=0.9307, p=0.5082<br>Two way RM ANOVA	P = 0.9976
**S7A**	Mean spike # CA1<br>(25Hz train)	BL vs<br>DMSO		7 slices	BL vs DMSO:<br>Interaction: $F_{(9, 54)}$=1.027, p=0.4310<br>Group: $F_{(1, 6)}$=1.000, p=0.3559<br>Time: $F_{(9, 54)}$=3.038, p=0.0052<br>Two way RM ANOVA	P = 0.9878
**S7C**	Mean # CA1 spikes	BL vs<br>NAB14 or<br>DMSO	BL: 2.02 ± 0.4<br>NAB: 2.33 ± 0.5<br>BL: 2.25 ± 0.4<br>DMSO: 2.06 ± 0.4	NAB: 9<br>slices<br>DMSO:<br>7 slices	NAB14: p=0.1007<br>Paired Student's t test<br>DMSO: p=0.3125<br>Wilcoxon matched-pairs	GH: p=0.8489<br>SH: n/a
**S7D**	Mean 1st spike latency<br>CA1	BL vs<br>NAB14 or<br>DMSO	BL: 19.22 ± 1.0<br>NAB: 18.52 ± 1.0<br>BL: 21.56 ± 0.7<br>DMSO: 21.01 ±0.8	NAB: 9<br>slices<br>DMSO:<br>7 slices	NAB14: p=0.0078<br>DMSO: p=0.5000<br>Wilcoxon matched-pairs	GH: p=0.0066<br>SH: n/a
**S7E**	Mean 1st spike "jitter"<br>CA1	BL vs<br>NAB14 or<br>DMSO	BL: 3.25 ± 0.4<br>NAB: 2.27 ± 0.6<br>BL: 3.45 ± 0.5<br>DMSO: 3.55 ± 0.6	NAB: 9<br>slices<br>DMSO:<br>7 slices	NAB14: p=0.9796<br>Paired Student's t test<br>DMSO: p>0.9999<br>Wilcoxon matched-pairs	GH: p=0.6720<br>SH: n/a
**S7F**	Mean CA1 output<br>frequency	BL vs<br>NAB14 or<br>DMSO	BL: 276 ± 10<br>NAB: 291 ± 13<br>BL: 282 ± 7<br>DMSO: 273 ± 1	NAB: 9<br>slices<br>DMSO:<br>6 slices	NAB14: p=0.0616<br>Paired Student's t test<br>DMSO: p>0.9999<br>Wilcoxon matched-pairs	GH: p=0.3071<br>SH: n/a

Supplemental Table 2. Properties of LPP-evoked CA1 spiking recorded from slices derived from GH and SH mice.

**CA1 spike properties**	GH (n=25 slices)	SH (n=25 slices)
# of spikes	2.6 ± 0.2	3.1 ± 0.3
CV # of spikes	48 ± 3	48 ± 6
Mean max # of spikes	4.9 ± 0.3	5.2 ± 0.4
1st spike latency (ms)	20.0 ± 0.9	19.6 ± 0.6
"jitter" 1st spike latency (ms)	4.2 ± 0.4	3.9 ± 0.3
1st spike amplitude (□V)	86.8 ± 4.4	86.7 ± 5.1
% responses w. spikes	93.7 ± 1.4	90.5 ± 3.0
% responses w. ≥2 spikes	74.5 ± 4.8	73.0 ± 6.4
Instantaneous frequency (Hz)	260.6 ± 7.3	275.7± 10.1

Supplemental Table 3. Properties of LPP-evoked CA3 spiking recorded from slices derived from GH and SH mice.

**CA3 spike properties**	GH (n=14 slices)	SH (n=17 slices)
# of spikes	4.8 ± 0.24	5.3 ± 0.5
Mean max # of spikes	8.1 ± 0.5	8.2 ± 0.6
1st spike latency (ms)	8.6 ± 0.5	7.9 ± 0.5
"jitter" 1st spike latency (ms)	2.6 ± 0.3	3.9 ± 0.3
1st spike amplitude (☐V)	111.4 ± 15.6	103.4 ± 7.5
% responses w. spikes	99.8 ± 0.2	99.8 ± 0.2
% responses w. ≥2 spikes	99.5 ± 0.3	98.2 ± 0.9
Instantaneous frequency (Hz)	267.9 ± 12.5	263.4 ± 12.6
